# Platelet-rich plasma for patellar tendinopathy: a randomized controlled trial correlating clinical outcomes and quantitative imaging

**DOI:** 10.1093/radadv/umae017

**Published:** 2024-07-08

**Authors:** Rianne A van der Heijden, Zachary Stewart, Robert Moskwa, Fang Liu, John Wilson, Scott J Hetzel, Darryl Thelen, Bryan Heiderscheit, Richard Kijowski, Kenneth Lee

**Affiliations:** Department of Radiology, University of Wisconsin-Madison, Madison, WI 53792, United States; Department of Radiology & Nuclear Medicine, Erasmus University Medical Center, Rotterdam 3015 GD, The Netherlands; Department of Radiology, Massachusetts General Hospital/Harvard Medical School, Boston, MA 02115, United States; Department of Medical Physics, University of Wisconsin-Madison, Madison, WI 53792, United States; Department of Radiology, Massachusetts General Hospital/Harvard Medical School, Boston, MA 02115, United States; Department of Orthopedics and Rehabilitation, University of Wisconsin, Madison, WI 53792, United States; Department of Biostatistics and Medical Informatics, University of Wisconsin-Madison, Madison, WI 53792, United States; Department of Biomedical Engineering, University of Wisconsin-Madison, Madison, WI 53792, United States; Department of Orthopedics and Rehabilitation, University of Wisconsin, Madison, WI 53792, United States; Department of Radiology, New York University Grossman School of Medicine, New York, NY 10016, United States; Department of Radiology, University of Wisconsin-Madison, Madison, WI 53792, United States

**Keywords:** patellar tendinopathy, platelet-rich plasma, quantitative, shear wave elastography, MRI ultrashort TE, needle tenotomy

## Abstract

**Background:**

Patellar tendinopathy is a common overuse injury in active individuals, often with incomplete recovery. Recently, platelet-rich plasma (PRP) treatment has shown promising results. Traditional qualitative markers are not reliable indicators of treatment response. Advanced quantitative imaging, such as ultrashort-echo time (UTE) MRI and US shear-wave elastography may be valuable adjuncts.

**Purpose:**

To investigate the clinical outcomes and quantitative imaging changes in adults with symptomatic patellar tendinopathy treated with PRP, needle tenotomy, or sham injection.

**Materials and Methods:**

Single-blinded prospective randomized controlled trial from April 2017 until July 2022 with 3 parallel interventions in athletes with symptomatic patellar tendinopathy: PRP, needle tenotomy, and sham injection. Visual analog scale for pain, patella tendinopathy-specific Victorian Institute of Sport Assessment Patella function, conventional US, shear wave speed (SWS), UTE T2* relaxation time (T2*_single_), and T2* fraction of fast-relaxing macromolecular-bound water were acquired at 0, 16 and 52 weeks. Longitudinal analyses were used to compare intra- and inter-group differences over time. Correlations were assessed by Pearson’s correlation coefficient.

**Results:**

A total of 29 subjects (mean age, 26.1 ± 5.3 years; 82.8% men) were randomized. At 52 weeks, all groups demonstrated a significant improvement in pain, though most pronounced within the PRP group (Δvisual analog scale = –5.9, 95% confidence interval [–7.8 to –3.9], *P* < .001). SWS increased significantly only in the PRP group (Δ+2.3 [0.8-3.9], *P* = .003). Change in SWS was moderately correlated with change in pain across all groups (*r* = –.52 [–.76 to –.15], *P* = .009). T2* fraction of fast-relaxing macromolecular-bound water significantly increased in all groups (Δ = 0.10-0.11, *P* = .024-0.046); a significant decrease in T2*_single_ was only seen in the PRP group (Δ = -8.07 [–14.6 to –1.55], *P* = .014).

**Conclusion:**

Clinical improvement was evident regardless of treatment but was greatest with PRP. SWS correlated with improvement in pain and may represent an adjunctive measure to assess healing in patellar tendinopathy. Correlative changes in T2* UTE quantitative markers suggest their potential for response assessment, but further research is needed to clarify their clinical applicability.

**Summary**

Platelet-rich plasma injection improved pain in patellar tendinopathy over a 1-year period compared to needle tenotomy and sham, with treatment response also assessed with quantitative ultrasound and MRI.

**Key Results**

- There was clinical improvement at 52 weeks across treatment groups irrespective of intervention, with the largest improvement when platelet-rich plasma (PRP) was applied.
- At 52 weeks, there was a moderate correlation between the change in US shear wave elastography speeds and pain improvement across all treatment groups.
- At 52 weeks, the fraction of bound water seen on ultrashort echo time MRI increased significantly across all treatment groups, whereas a significant decrease in T2*_single_ was only seen with PRP treatment.

## Introduction

Patellar tendinopathy (PT) is a common condition primarily affecting active individuals engaged in jumping activities.^1^ PT is characterized by pain at the proximal patellar tendon and reduced knee function.^2^ Presumed etiology is overuse-induced degeneration and inflammatory changes in the patellar tendon.^3^ Structural changes include collagen disorganization, increased proteoglycan and glycosaminoglycan, increased free bulk water, and decreased macromolecular bound water and neovascularization.^4–6^ Conservative treatment is not consistently successful, which has led to exploration of minimally invasive interventions. Many of these interventions, including platelet-rich plasma injection (PRP) and needle tenotomy (NT), address the structural changes through recruitment of growth factors to the site of the injury.^7^

Imaging is important for diagnosing PT, but assessment of treatment response remains challenging. Conventional qualitative imaging techniques provide subjective information but do not always correlate with outcomes.^8^ Quantitative US and MRI techniques, such as shear wave elastography (SWE) and ultrashort echo time (UTE) T2* MRI, have shown potential for objectively assessing tendinopathy.^9–19^

SWE uses an acoustic radiation force pulse to generate shear waves perpendicular to the US beam causing transient tissue displacements. The corresponding shear-wave speeds (SWS) are directly related to the shear modulus, an absolute measure of the tissue’s elastic properties. SWS are lower in disorganized tendons than in normal compact fibrillar ones.^17^ Previous literature has suggested that SWS may be a more objective and sensitive marker for tissue healing than conventional US.^10^

UTE-MRI visualizes tissues by capturing the rapidly decaying signal undetectable with conventional T2 imaging with UTE.^20^ UTE-T2* relaxation time can be quantified by applying a single-component or a bicomponent model. In prior studies, decreases in single-component T2* relaxation time (T2*_single_) have been correlated with improved clinical outcome in PT after treatment.^1^ However, T2*_single_ is a nonspecific parameter, averaging the relaxation characteristics of the individual water components of the tissue. Bicomponent UTE-T2* mapping may provide more specific information of both macromolecular water-bound and free water components in tendon healing.^11–16^^,^^21^

This single-blinded randomized controlled trial (RCT) investigated the relative effectiveness of 3 parallel PT interventions, PRP, NT, or sham injection (SH), as measured by patient-reported outcomes (PROs) with a 52-week follow-up. In addition, this study investigated the role of quantitative imaging parameters—US SWS and MRI UTE-T2* single-component and bicomponent parameters—as markers of patellar tendon healing.

## Materials and methods

### Trial design

The study was a single-blinded multiarm parallel group RCT comprising 3 interventions for PT (PRP, NT, and SH). Subjects were recruited and block randomized by the study nurse using an allocation ratio of 1:1:1. Subjects were blinded to treatment group and blood was drawn in all participants. A musculoskeletal radiologist (K.L.) with 20 years of experience performed all interventions. PRP included single US-guided injection of 5 mL autologous leukocyte-rich PRP (Arthrex ACP Double Syringe System, Arthrex Inc., Naples, FL) with a 22G, 1.5-inch needle within the abnormal tendon and the enthesis after 10 needle passes. NT included 20 passes with a 22G, 1.5-inch needle targeting hypoechoic and hyperemic tendon. SH included 20 needle passes only in the subcutaneous tissues superficial to the patella tendon. Two blinded independent researchers (R.v.d.H., Z.S.) postprocessed all data. An independent statistician (S.H.) performed all statistical analyses.

### Participants

Institutional review board and written informed consent were obtained, and the Health Insurance Portability and Accountability Act was adhered to. Patients with PT were diagnosed by a sports medicine specialist (J.W.) with 17 years of experience at the University Hospital Orthopedics Clinics. Inclusion criteria included: age 18-39 years, chronic (>3 months) PT, MRI, or US evidence of PT, visual analog scale (VAS) pain score of >3, self-reported failure of >2 conservative treatments (eg, nonsteroidal antiinflammatory drugs, relative rest, ice, bracing) and supervised physical therapy. Exclusion criteria included: inability to comply with follow-up, hematologic conditions, knee pain from other etiologies (eg, degenerative joint disease, trauma), full or partial patellar tendon tear, use of anticoagulation or immunosuppression, prior surgery, pregnancy, worker’s compensation injury, daily opioid use, MRI contraindication, systemic diseases such as diabetes or connective tissue diseases and prior PRP or NT procedure.

### Clinical outcome, US, and UTE MRI measures

VAS during activity, PT-specific Victorian Institute of Sport Assessment Patella (VISA-P) score, and Tegner Activity Scale were collected at baseline, 16 weeks, and 52 weeks.^22^^,^^23^

Both conventional (B-mode and power-Doppler) and quantitative (SWE) US images were obtained by a single operator (K.L.) at all timepoints using a linear high frequency (6-15 MHz) transducer (Supersonic Imagine, Aix-en-Provence, France) with a gel stand-off pad in long axis to the patella tendon with the knee in 30° flexion as measured by a goniometer.

A sagittal 3-dimensional multi-echo UTE-T2* sequence (3D-Cones, GE Healthcare, Waukesha, WI) of the knee was performed at all time points using a 3.0 T scanner (Discovery MR750, GE Healthcare) and 8-channel phased-array extremity coil (InVivo, Orlando, FL). The sequence used a 3D cone k-space sampling scheme with 16 echo times between 0.003 ms and 35 ms. Additional parameters included a 40 ms repetition time, 20° flip angle, 16 cm field-of-view, ±150 KHz bandwidth, 256 × 256 matrix, 3-mm slice thickness, 1 excitation, 10 slices through the patellar tendon, and 19-minute scan time.

### Image postprocessing and analysis

**Figure 1.**
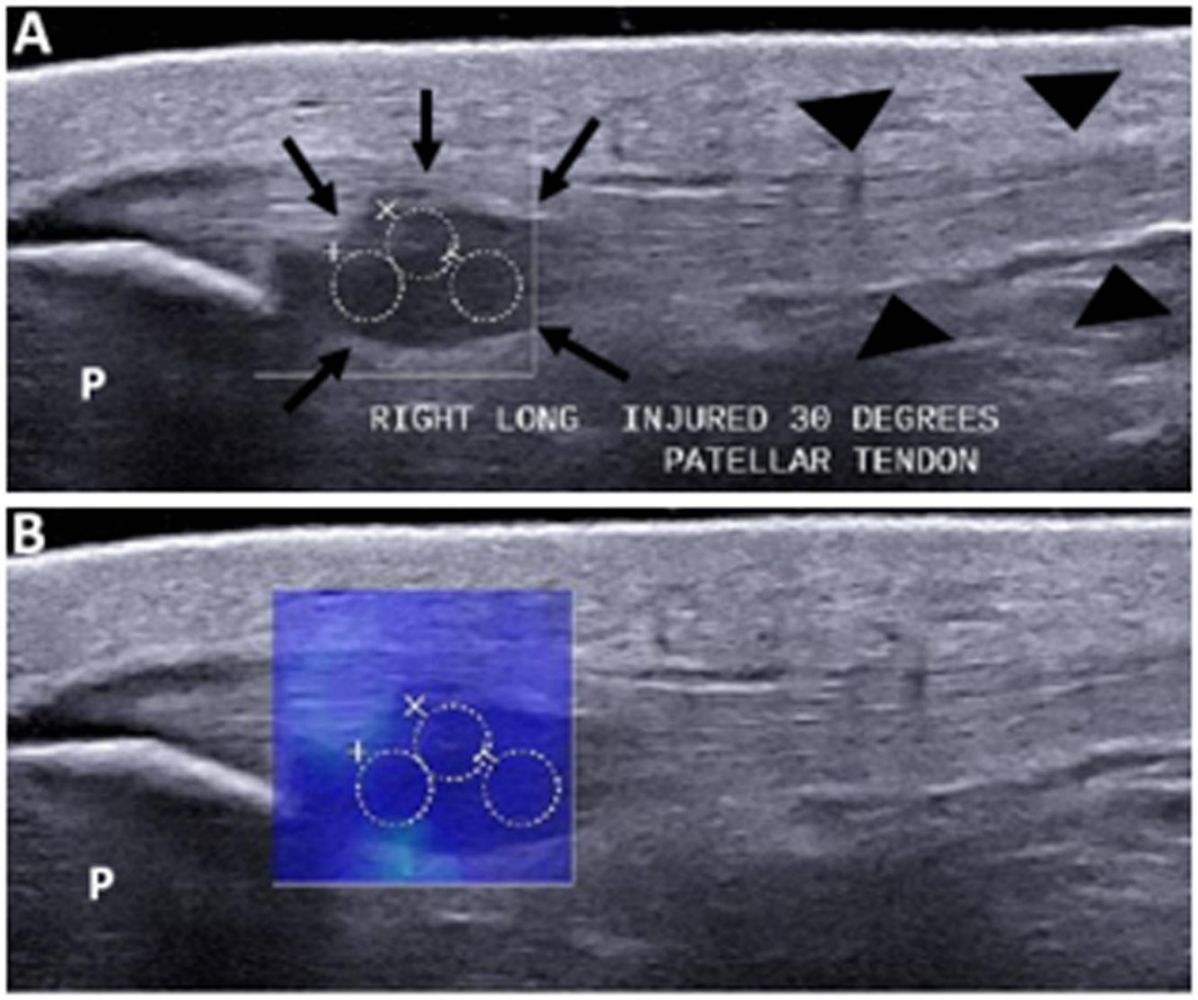
Example of the delineated region of interest (ROI) of the proximal patellar tendon on ultrasound gray scale long-axis image (A) and corresponding shear wave speed (SWS) color-map image (B). The ROIs (circles) are placed on the affected/hypoechoic portion of the proximal patella tendon (arrows), near the attachment to the inferior pole of the patella (P). Notice the normal fibrillar and hyperechogenicity of the normal portion of the patella tendon distally (arrowheads).

A musculoskeletal radiologist with 5 years of experience (Z.S.) retrospectively reviewed all conventional and SWE US images. The thickest portion of the tendon (mm), and hypo-echogenicity and hyperemia (0 = normal to 3 = severe) were reported. The SWS maps had three 10-mm diameter regions of interest (ROIs) corresponding to diseased areas on the baseline B-mode images (Figure 1). SWS was averaged across the ROIs. Care was taken to measure SWS in the exact same location on follow-up.

**Figure 2.**
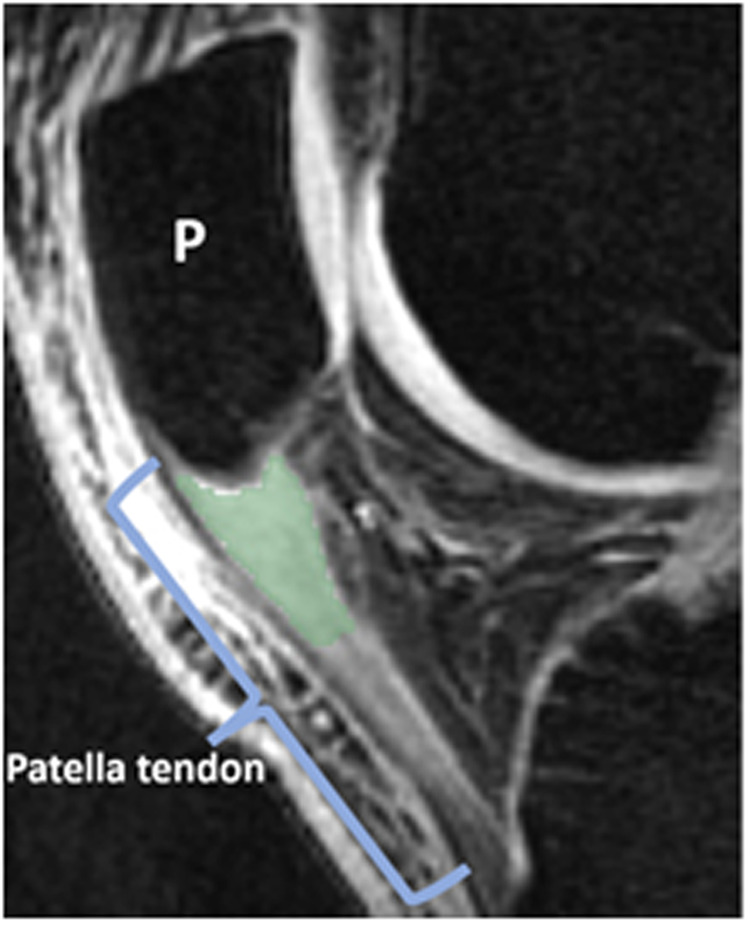
Example of the delineated region of interest (ROI) of the proximal patellar tendon on an ultrashort TE-T2* sagittal magnetic resonance image. The color shading delineates the ROI placed on the proximal patella tendon near its attachment to the patella (P).

For MRI, a musculoskeletal radiologist with 6 years of experience (R.v.d.H.) delineated ROIs of the proximal 1 cm of the patellar tendon on TE 4.3 ms UTE image using 3D Slicer (v5.6.1, Brigham and Women’s Hospital, Harvard Medical School, Boston, MA, USA) (Figure 2). All echoes were registered using rigid registration.^24^ Single-component T2*_single_ and bicomponent fast-relaxing macromolecular-bound water component (T2*_fast_), slow relaxing bulk water component (T2*_slow_), and fraction of fast-relaxing macromolecular-bound water component (F_F_) parameter maps were calculated by voxel-by-voxel curve fitting using MATLAB (MATLAB 2010b, MathWorks Inc, Natick, MA).^21^ After superimposing the ROI over the UTE-T2* parameter maps, mean and SD values were obtained. Excellent repeatability has been demonstrated in a prior study.^13^

### Statistics

The sample size was calculated for comparing VISA-P score change from baseline to 52 weeks between groups. With an expected Cohen’s D effect size of 0.52, 20 subjects per group were estimated to result in a 95% power. With 29 total subjects prior to analysis, there would be 65% power to detect an effect size of 0.52 and 80% power to detect a slight larger, but still attainable, effect size of 0.61. All analyses followed intention-to-treat. Missing data were treated as missing at random.

Longitudinal data analysis with subject as a random effect were used to compare between-group and within-group differences in mean outcome measures over time. Group allocation, a 3-level factor for time, and their interaction term were fixed predictors in the longitudinal data analysis models. Pearson’s correlation was used to assess correlations of change from baseline to 52 weeks between outcome measures. To account for multiple comparisons, a Tukey’s family-wise adjustment was done in the case of a post hoc examination of the 2-way comparison. *P* values <.05 were considered significant. All statistical analyses were performed using R Statistical Software (v4.0; R Core Team 2020; Vienna, Austria).

## Results

### Study population

**Figure 3.**
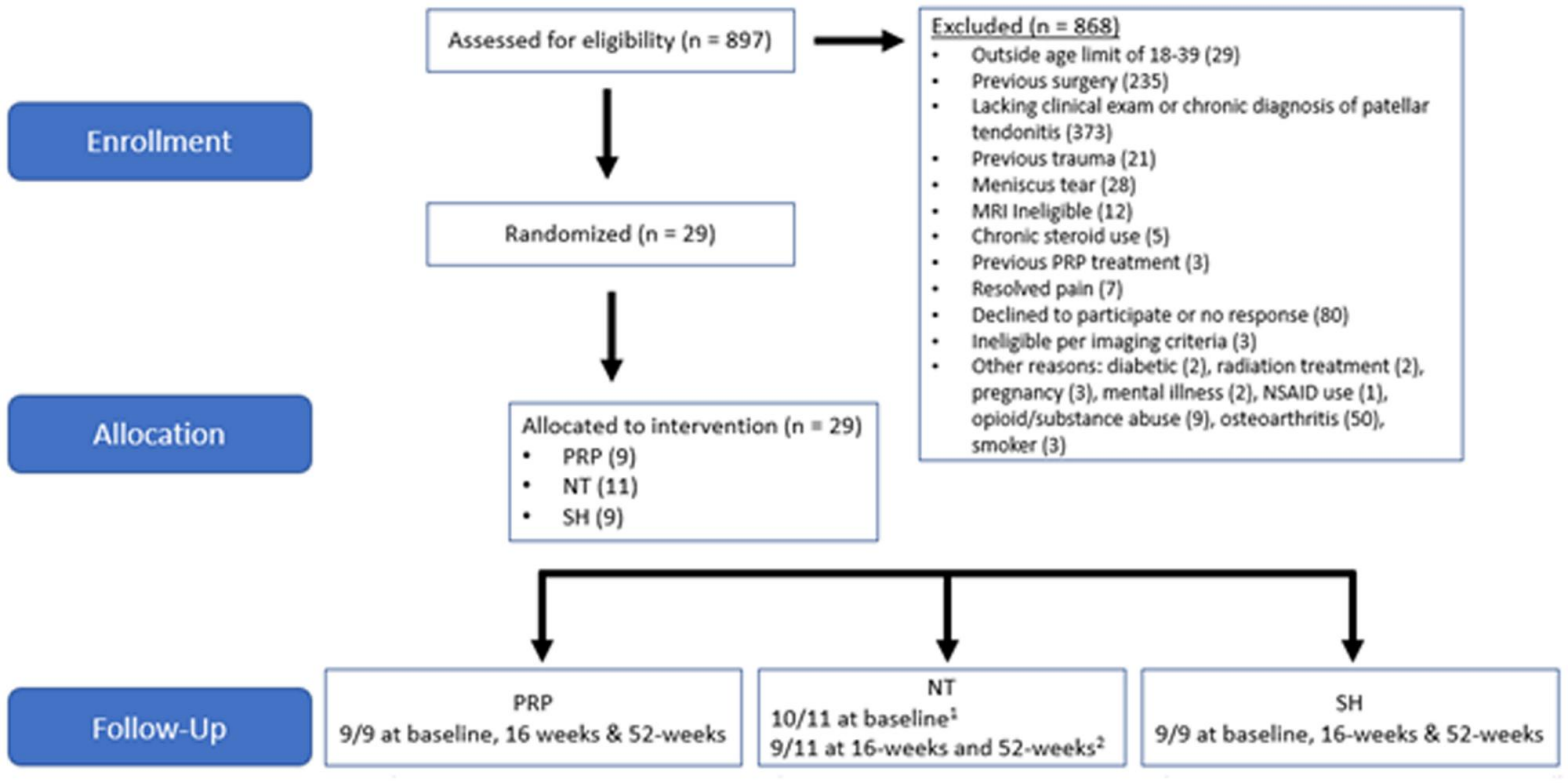
Flowchart of patient inclusion. Abbreviations: PRP = platelet-rich plasma, NT = needle tenotomy, SH = sham. ^a^Patient symptoms and imaging evidence of patellar tendinopathy resolved between enrollment and baseline visit. ^b^Patient lost to follow-up after baseline visit.

A total of 897 patients were screened, and ultimately 29 athletes were enrolled between April 2017 and July 2022 and randomized into the following groups: PRP = 9; NT = 11; and SH = 9 (Figure 3, Table 1). One patient in the NT group did not receive the intervention because the patient’s symptoms and US-imaging evidence of PT resolved between enrollment and their baseline visit. One patient from the NT group was lost to follow-up after the baseline visit. For the MRI analyses, 1 patient in the SH group was excluded because of mid-tendon involvement rather than proximal tendon. Three patients (1 PRP, 2 NT) were excluded from the MRI analysis for varying technical reasons such as failed registration and artifacts. MRI data were available for 19 patients at baseline (6 SH, 5 NT, 8 PRP), 20 patients at 16 weeks (7 SH, 7 NT, 6 PRP), and for 16 patients at 52 weeks (6 SH, 6 NT, 4 PRP). No adverse events were reported.

**Table 1.** Patient characteristics.

	Total	PRP	Needle tenotomy	Sham	*P*-value
**N**	29	9	11	9	
**Age,** mean (SD)	26.1 (5.3)	25.7 (4.9)	26.6 (5.6)	25.8 (5.9)	.908
**BMI,** mean (SD)	26.4 (5.0)	26.9 (5.8)	25.2 (2.9)	27.4 (6.3)	.604
**Sex (M: F)**	24 (83%): 5 (17%)	8 (89%): 1 (11%)	9 (82%): 2 (18%)	7 (78%): 2 (22%)	1
**Race (self-reported)**					
African-American (Black)	1	1 (11.1%)	0 (0.0%)	0 (0.0%)	
American Indian or Alaska Native	1	0 (0.0%)	0 (0.0%)	1 (11.1%)	
Caucasian	27	8 (88.9%)	11 (100.0%)	8 (88.9%)	

Abbreviation: PRP = platelet rich plasma.

The patients in the 3 treatment arms were not statistically different with respect to age, body mass index, sex, and race. Most participants were Caucasian.

### Outcome

The PRP and SH groups demonstrated significant improvements in VAS pain during activity at 16 weeks (Table 2). At 52 weeks, all groups demonstrated significant improvements in VAS pain during activity with this effect appearing most pronounced in the PRP group (Table 2). At 16 weeks, the VISA-P score improved significantly for the PRP group (20.3, 95% confidence interval [CI, 5.0-35.7], *P* = .006). At 52 weeks, the VISA-P score improved statistically significantly for the PRP and SH groups (Table 2). On the Tegner Activity Scale, only the PRP group demonstrated a significant improvement at 52 weeks (Table 2).

**Table 2.** Clinical outcome at baseline, week 16, and week 52 across the 3 treatment groups.

	Week 0	Week 16	Week 52	Week 16-0	*P*-value	Week 52-0	P-value
**VAS** (0-10)							
PRP	7.4 (5.9-9.0)	4.1 (2.5-5.6)	1.6 (0.0-3.2)	–3.39 (–5.29 to –1.49)	**<.001**	–5.87 (–7.84 to –3.89)	**<.001**
NT	6.9 (5.5-8.3)	5.7 (4.2-7.2)	4.5 (2.8-6.1)	–1.24 (–3.09 to 0.62)	.26	–2.45 (–4.47 to –0.43)	**.01**
SH	7.7 (6.1-9.2)	4.4 (2.9-6.0)	3.3 (1.8-4.9)	–3.22 (–5.13 to –1.32)	**<.001**	–4.33 (–6.24 to –2.43)	**<.001**
Group level	*P* = .39						**.04**
**VISA-P** (0-100)							
PRP	45.0 (32.2-57.8)	65.3 (52.5-78.1)	76.8 (64.0-89.6)	20.3 (5.0-35.7)	**.006**	31.8 (16.4-47.1)	**<.001**
NT	57.9 (46.3-69.5)	69.4 (56.9-82.0)	69.3 (55.6-83.0)	11.5 (–3.46 to 26.5)	.17	11.4 (–4.9 to 27.7)	.23
SH	56.0 (43.2-68.8)	66.6 (53.7-79.4)	73.7 (60.3-87.0)	10.6 (–4.78 to 25.9)	.24	17.7 (1.7-33.6)	**.03**
Group level	*P* = .23						.15
**Tegner Activity Scale** (1-10)							
PRP	4.0 (2.8-5.2)	4.7 (3.5-6.0)	6.1 (4.8-7.3)	0.72 (–0.71 to 2.15)	.478	2.06 (0.63-3.48)	**.003**
NT	6.1 (5.0-7.2)	5.7 (4.5-6.9)	6.6 (5.2-7.9)	–0.38 (–1.77 to 1.02)	.82	0.47 (–1.06 to 1.99)	.78
SH	6.6 (5.3-7.8)	5.9 (4.6-7.1)	6.4 (5.2-7.7)	–0.67 (–2.10 to 0.76)	.54	–0.11 (–1.54 to 1.32)	.99
Group level	PRP vs NT ***P* = .04**; PRP vs SH ***P* = .02**						.32

Reported as estimated mean (95% CI) from longitudinal data analysis with subject as a random effect.

Abbreviations: NT = needle tenotomy, PRP = platelet-rich plasma, SH = sham, VISA-P = PT-specific Victorian Institute of Sport Assessment Patella, VAS = visual analog scale.

### Conventional US and SWE measurements

**Figure 4.**
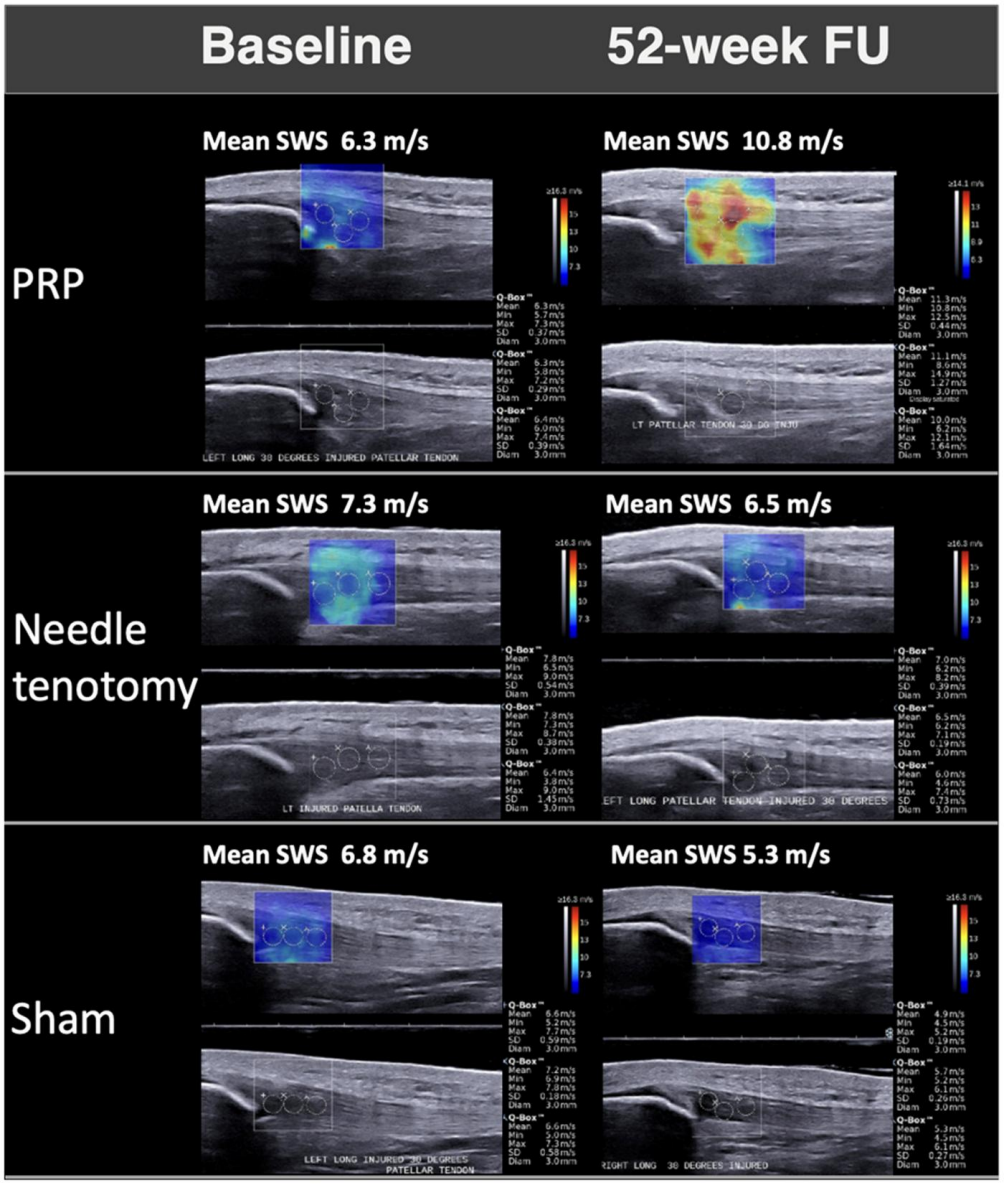
Sagittal ultrasound shear wave speed (SWS) maps at baseline (left) and 52-week follow-up (right) in 3 patients with patellar tendinopathy (26-year-old male, 25-year-old male, 20-year-old female) who underwent platelet-rich plasma (PRP), needle tenotomy, and sham treatment, respectively. After PRP treatment, statistically significant improvement in SWS from 6.3 to 10.8 m/s was demonstrated, whereas the needle tenotomy and sham treatment showed no improvement over time.

**Figure 5.**
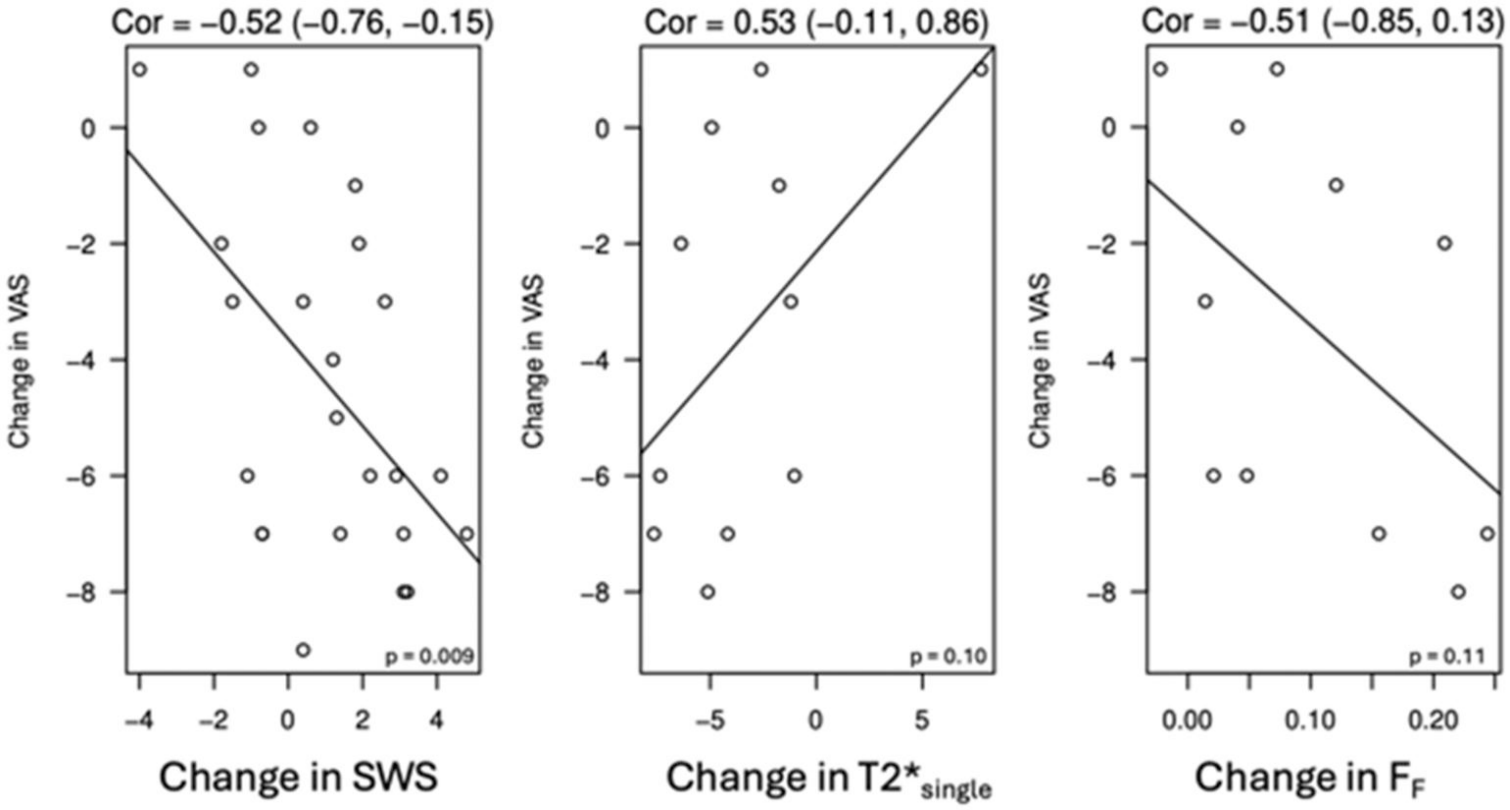
Plots showing correlations between change in visual analogue scale (VAS) pain score and changes in imaging values on ultrashort echo time MRI and ultrasound shear wave elastography at week 52. Abbreviations: F_F_ = fraction of the fast-relaxing macromolecular bound water component, SWS = shear wave speed on ultrasound, T2*single = single-component UTE-T2* relaxation time.

At baseline, the NT group had a significantly smaller tendon thickness (*P* = .027) and higher SWS (*P* = .025) than the SH group. The baseline conventional and qualitative US parameters were otherwise not significantly different among the treatment groups at baseline. At 16 weeks, there was no significant change in the US parameters in any of the treatment groups. At 52 weeks, only the SH group experienced a significant change in the conventional US parameters with a decrease in both tendon thickness and hyperemia. There was no change in the hypo-echogenicity in any of the treatment groups at 52 weeks (Table S1). There was a significant increase in SWS in only the PRP group at 52 weeks (Table 3, Figure 4). The change in conventional US measures was not significantly correlated with the change in VAS (Table 4). The change in SWS at 52 weeks was moderately correlated with the change in VAS (–0.52 [–0.76 to –0.15] *P* = .009) (Figure 5).

**Table 3.** Quantitative imaging values on ultrashort TE MRI and ultrasound elastography for baseline, week 16, and week 52 across the 3 treatment groups.

	Week 0	Week 16	Week 52	Week 16-0	*P*-value	Δ Week 52-0	*P*-value
T2*_single_ (ms)							
PRP	15.2 (11.8, 18.6)	11.7 (7.7, 15.7)	7.1 (2.3, 12.0)	−3.5 (-9.2, 2.2)	.28	−8.1 (-14.6, -1.6)	**.010**
NT	10.6 (6.3, 15.0)	8.7 (5.0, 12.4)	6.9 (2.9, 10.9)	−1.9 (-8.2, 4.3)	.68	−3.7 (-10.2, 2.8)	.32
SH	14.9 (11.0, 18.9)	13.4 (9.7, 17.1)	11.7 (7.7, 15.7)	−1.6 (-7.5, 4.3)	.75	−3.2 (-9.3, 2.9)	.37
*group-level*	*P* = .47						.75
**F_F_ (%)**							
PRP	0.5 (0.5, 0.6)	0.6 (0.5, 0.6)	0.6 (0.5, 0.7)	0.02 (-0.06, 0.11)	.74	0.10 (0.00, 0.20)	**.046**
NT	0.6 (0.5, 0.6)	0.6 (0.5, 0.7)	0.7 (0.6, 0.7)	0.03 (-0.07, 0.12)	.75	0.11 (0.01, 0.21)	**.030**
SH	0.4 (0.4, 0.5)	0.5 (0.4, 0.6)	0.5 (0.5, 0.6)	0.07 (-0.02, 0.16)	.17	0.10 (0.01, 0.20)	**.020**
*group-level*	*P* = .13						.89
T2*_fast_ (ms)							
PRP	2.2 (1.7, 2.7)	1.9 (1.3, 2.5)	2.1 (1.4, 2.8)	−0.26 (-1.02, 0.50)	.64	−0.06 (-0.95, 0.82)	.97
NT	1.8 (1.1, 2.4)	2.1 (1.5, 2.7)	2.6 (2.0, 3.2)	0.33 (-0.52, 1.18)	.57	0.82 (-0.07, 1.71)	.07
SH	2.9 (2.3, 3.5)	2.6 (2.0, 3.2)	2.5 (2.0, 3.1)	−0.27 (-1.06, 0.53)	.65	−0.32 (-1.14, 0.49)	.55
*group-level*	NT vs SH ***P* = .03**						.27
T2*_slow_ (ms)							
PRP	30.0 (24.6, 35.3)	27.6 (21.4, 33.7)	28.6 (21.2, 36.1)	−2.4 (-10.6, 5.8)	.71	−1.3 (-10.8, 8.2)	.91
NT	27.9 (21.2, 34.6)	28.8 (23.0, 34.5)	33.2 (27.1, 39.4)	0.8 (-8.3, 9.9)	.96	5.3 (-4.2, 14.9)	.34
SH	28.4 (22.2, 34.6)	29.2 (23.5, 35.0)	28.6 (22.5, 34.8)	0.8 (-7.7, 9.4)	.95	0.2 (-8.6, 9.0)	.96
*group-level*	*P* = .92						.73
**SWS (m/s)**							
PRP	6.3 (5.1, 7.4)	7.3 (6.1, 8.4)	8.6 (7.4, 9.7)	1.0 (-0.56, 2.56)	.26	2.31 (0.75, 3.88)	**.003**
NT	7.6 (6.5, 8.6)	7.5 (6.3, 8.6)	7.0 (5.7, 8.3)	−0.09 (-1.60, 1.42)	.98	−0.60 (-2.24, 1.05)	.62
SH	5.7 (4.6, 6.9)	5.9 (4.7, 7.0)	6.4 (5.2, 7.6)	0.12 (-1.44, 1.69)	.97	0.67 (-0.90, 2.23)	.52
*group-level*	NT vs SH ***P* = .03**					PRP vs NT ***P* = .01**	.08

Reported as estimated mean (95% CI) from longitudinal data analysis with subject as a random effect.

Abbreviations: F_F_ = fraction of the fast-relaxing macromolecular bound water component, NT = needle tenotomy, PRP = platelet-rich plasma, SH = sham, SWS = shear wave speed on ultrasoundT2*fast = fast-relaxing macromolecular-bound water component, T2*single = single-component UTE-T2* relaxation time, T2*slow = slow relaxing bulk water component.

**Table 4.** Linear correlation of change from baseline to week 52 for quantitative MRI and US measures, conventional US measures, and patient-reported outcomes.

	Week 0	Week 16	Week 52	Week 16-0	*P*-value	Week 52-0	*P*-value
**VAS** (0 - 10)							
PRP	7.3 (5.7-8.8)	4.2 (2.6-5.8)	1.5 (–0.1to 3.2)	–3.06 (–4.86 to –1.26)	<.001	–5.70 (–7.58 to –3.82)	**<.001**
NT	6.8 (5.4-8.2)	5.4 (3.9-7.0)	4.9 (3.2-6.6)	–1.39 (–3.15 to 0.36)	.15	–1.86 (–3.80 to 0.08)	.06
SH	7.7 (5.8-9.5)	4.7 (2.8-6.5)	4.8 (3.0-6.7)	–3.00 (–5.08 to –0.92)	.003	–2.83 (–4.91 to –0.76)	**.005**
Group level	*P* = .36						**.007**
**VISA-P** (0-100)							
PRP	46.6 (32.7-60.5)	67.4 (53.5-81.3)	76.7 (62.9-90.6)	20.75 (3.89-37.61)	.012	30.12 (13.26- 46.99)	**<.001**
NT	58.9 (46.4-71.4)	69.5 (55.8-83.1)	67.9 (52.8-83.1)	10.60 (–5.81 to 27.00)	.29	9.03 (–9.08 to 27.15)	.48
SH	55.5 (39.4-71.6)	65.2 (49.0-81.3)	72.6 (55.4-89.8)	9.67 (–9.81 to 29.14)	.49	17.08 (–3.63 to 37.80)	.13
Group level	*P* = .36						.25
**Tegner Activity Scale** (1-10)							
PRP	3.9 (2.6-5.1)	5.1 (3.8-6.3)	5.7 (4.4-6.9)	1.19 (–0.18 to 2.55)	.1	1.81 (0.45-3.18)	**.006**
NT	5.7 (4.6-6.8)	5.4 (4.2-6.6)	6.3 (5.0-7.6)	–0.34 (–1.67 to 1.00)	.84	0.63 (–0.85 to 2.10)	.6
SH	6.0 (4.6-7.4)	5.7 (4.2-7.1)	6.3 (4.9-7.8)	–0.33 (–1.91 to 1.24)	.89	0.33 (–1.24 to 1.91)	.89
Group level	*P* = .04; no sig. 2-way comparison after adjustment						.25

Reported as Pearson correlation coefficient and 95% CI for change from baseline at week 52.

Abbreviations: F_F_ =fraction of the fast-relaxing macromolecular bound water component, SWS = shear wave speed on ultrasound, T2*fast =fast-relaxing macromolecular-bound water component, T2*single =single-component UTE-T2* relaxation time, T2*slow= slow relaxing bulk water component, VAS = visual analog scale, VISA-P = PT-specific Victorian Institute of Sport Assessment Patella.

### UTE MRI measurements

**Figure 6.**
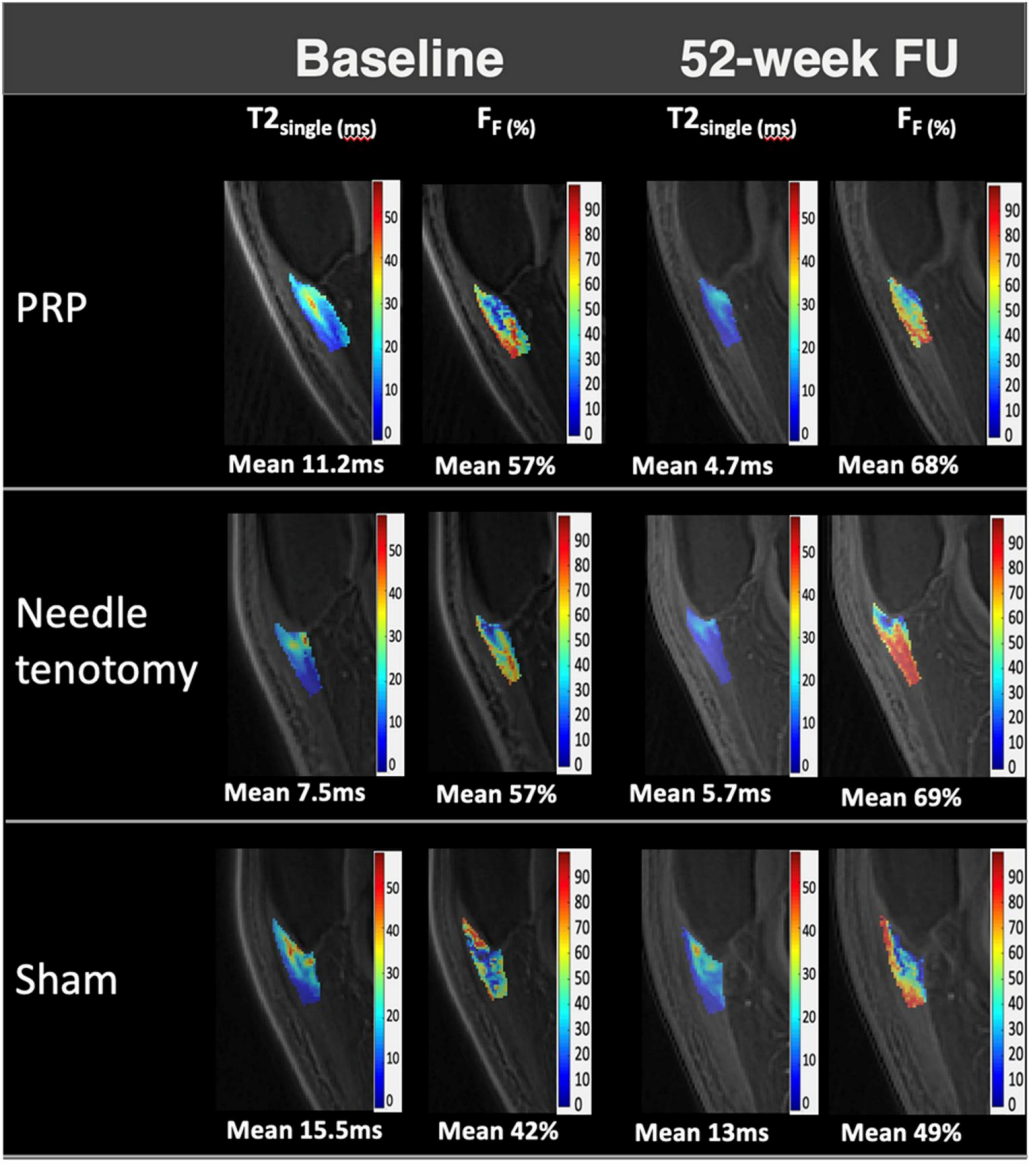
MRI T2*_single_ (ms) and F_F_ (%) parameter maps at baseline and 52-week follow-up in 3 patients with patellar tendinopathy (30-year-old male, 34-year-old male, 20-year-old male) following platelet-rich plasma (PRP), needle tenotomy, and sham treatment, respectively. Improvements in T2*_single_ and F_F were_ seen in all groups, but the greatest improvement was demonstrated in the PRP group. Abbreviations: F_F_ = fraction of the fast-relaxing macromolecular bound water component, T2*single = single-component UTE-T2* relaxation time.

The NT group had a significantly lower T2*_fast_ (*P* = .027) compared to the SH group at baseline (Table 3). The baseline quantitative MRI parameters were otherwise not significantly different among the treatment groups. F_F_ significantly increased from baseline to 52 weeks in all groups (Figure 6). Only the PRP group demonstrated a significant decrease in T2*_Single_ (Figure 6). There was no significant change in T2*_fast_ or T2*_slow_ in any of the groups at 52 weeks. The changes in T2*_single_ and F_F_ at 52 weeks were moderately correlated with the change in VAS, respectively, but the P values did not show statistical significance for these correlations (Table 4, Figure 5).

No correlations were demonstrated between either UTE measures or SWS and VISA-P. Also, no significant correlation was demonstrated between quantitative MRI parameters and SWS. F_F_ was significantly correlated with a decrease in hyperemia (–0.62 [–0.88 to –0.07] *P* = .030).

### Subgroup analysis

A total of 5 patients were affected by temporary (∼5-month) restriction of team sport activities relating to the COVID-19 pandemic.^25^ Of these 5 patients, 3 were in the SH cohort and 1 patient each from the NT and PRP cohorts. Subgroup analysis demonstrated that those unaffected by a temporary activity restriction related to the pandemic experienced a less dramatic decrease in VAS pain at 52 weeks (Table S2). This effect was primarily seen in the SH and NT cohorts and was negligible for the PRP cohort. In this subanalysis, the improvement in pain in the NT group was not statistically significant, the SH group maintained a statistically significant improvement with smaller effect size, and the effect in the PRP group was essentially unchanged with a significant reduction in VAS pain at 52 weeks.

## Discussion

The presented sham-controlled RCT has compared PRP to NT using multimodal imaging to assess tendon healing over 52 weeks. Improvement in VAS was demonstrated across all treatment groups at 52 weeks; however, only the PRP group demonstrated significant improvement in all PROs. There was a moderate correlation between the changes in SWS and VAS at 52 weeks across treatment groups. Only the PRP group showed a significant increase in SWS and decrease in T2*_single_ at 52 weeks, whereas all groups demonstrated an increase in F_F_ at 52 weeks.

A key finding was the improvement in VAS across all treatment groups, including SH, which suggests improvement without any minimally invasive treatment with rest.^26^ Activity restrictions imposed by the COVID-19 pandemic may have contributed to improvement in pain especially in the SH group, because the lockdown affected a larger proportion of subjects in that group. The placebo effect could also have contributed to these results.^27^

Among all treatment groups, the PRP cohort reported the greatest improvement in VAS and only the PRP cohort reported significant clinical improvement in all PROs at 52 weeks. The role of PRP in PT and its relative effectiveness to other interventions are still unclear. A 2022 meta-analysis found that PRP injections did not provide clinical benefit in terms of pain relief compared to other non-PRP injection alternatives.^28^ Additionally, a 2021 systematic review and meta-analysis concluded that there was no “high-quality evidence to convincingly demonstrate superior outcomes with specific treatment modalities (for patella tendinopathy).”^29^

Current conventional qualitative imaging does not always correlate with clinical outcomes. Prior studies have suggested that ultrasound SWE may serve as a quantitative imaging metric to assess tendon healing.^17^^,^^18^^,^^30^ In this study, increase in SWS was correlated with improvement in PT-related pain, adding further credence to this notion. Interestingly, no significant correlation was found between the changes in SWS and the other PROs.

With regard to UTE T2* parameters, F_F,_ increased significantly across all groups at 52 weeks, whereas T2*_single_ only decreased significantly in the PRP group and no significance was demonstrated for T2*_fast_, T2*_slow._ The significant changes in F_F_ were driven by both a decrease in bulk water and an increase in macromolecular bound water because of macromolecular matrix repair during tendon healing.^31^ The difference between single- and bicomponent UTE is probably because T2*_single_, a composite measure of bicomponent parameters T2*_fast_, T2*_slow_, and the weighting factor fraction of bound water F_F_, is less specific to changes in the individual water compartments. These results were in concordance with prior literature showing that the fraction of free and bound water was more sensitive than T2*_single_, T2*_fast_, and T2*_slow_ for detecting compositional and structural changes in articular cartilage degeneration^32^ and tendinopathy.^13^

There were moderate correlations between VAS at 52 weeks and T2*_single_ and F_F_, respectively, but P values were above the threshold for statistical significance. Other studies have reported correlations between PROs and UTE-MRI.^12^ It is possible that the results in this study were limited by the small sample size.

There was no correlation between SWS and F_F,_ or T2*_single_. This may be due to a lack of power or due to a true difference between imaging parameters. SWS reflects tendon elastic properties, which is primarily dependent on collagen fiber organization and macrostructure, whereas UTE-MRI may be more reflective of tendon biochemical composition and microstructure.^12^^,^^33^ F_f_ may be a more sensitive measure of clinical improvement—because an increased F_F_ was seen across all groups, just as improvement in VAS. SWS could be a direct reflection of more robust or organized healing—because significant changes in SWS were only seen in the PRP group at 52 weeks. Quantitative US and MRI may serve as complementary, rather than interchangeable, modalities.

This study has several limitations. The COVID-19 pandemic limited recruitment and may have affected the participants’ activity level, indirectly affecting their clinical outcomes, especially in the SH group. Subgroup analysis of participants not being affected by temporary restriction of team sport activities, which may not be adequately powered, has been performed. The study was also limited by the small sample size and inability to acquire a substantial number of UTE-MRI follow-up data points. This precluded statistical adjustments for confounders and the powering necessary to find statistical significance for the correlations between the UTE-MRI parameters and PROs. The missing UTE-MRI data were equally distributed across groups, and the missing data points were used rather than imputing a large percentage of the data. A single operator performed all SWS US measurements. This was by design, as previous studies have shown good intra-rater reliability, whereas inter-rater reliability is less reliable.^34^^,^^35^ This design, however, did limit the ability to perform any inter-rater reliability measures for SWS. Finally, the study was limited by demographic homogeneity.

In summary, this study suggests that improvement in pain may be the natural course of PT with rest and physical therapy. However, the greatest degree of clinical improvement was with PRP treatment when compared to NT and SH. Additionally, this study demonstrated that quantitative imaging may have a role as an adjunct measure of tendon healing. Most notably was the correlation between improvement in pain and an increase in SWS. Potential has been shown for UTE imaging, but studies with a larger sample size are needed to further validate its clinical applicability. The promising results of this study may form the basis for future larger and more diverse clinical trials necessary to elucidate the clinical role of emerging minimally invasive treatments and quantitative imaging tools.

## Supplementary Material

umae017_Supplementary_Data

## Data Availability

NCT03136965. Data generated or analyzed during the study are available from the corresponding author by request.

## Abbreviations

NT: needle tenotomy; PRO = patient-reported outcome
PRP: platelet-rich plasma
PT: patellar tendinopathy
RCT: randomized controlled trial
SH: sham
SWE: shear-wave elastography
SWS: shear wave speed
UTE: Ultrashort echo time
VAS: visual analog scale
VISA-P: patella tendinopathy-specific Victorian Institute of Sport Assessment Patella
