# Supplementary material for: Platelet-rich plasma for patellar tendinopathy: a randomized controlled trial correlating clinical outcomes and quantitative imaging
Source: Radiol Adv. 2024 Jul 8;1(2):umae017. doi: 10.1093/radadv/umae017 (PMC12481695; doi:10.1093/radadv/umae017)
Supplement: umae017_Supplementary_Data [file umae017_Supplementary_Data.zip › Supplemental Tables for publication.docx]

**Supplemental Tables**

**Table S1.** Conventional ultrasound measurements for baseline, week 16, and week 52 across the three treatment groups. Reported as estimated mean (95% CI) from longitudinal data analysis with subject as a random effect. PRP=platelet-rich plasma, NT=needle tenotomy, SH=sham.

|  | **Week 0** | **Week 16** | **Week 52** | **Week 16-0** | **P-value** | **Δ Week 52-0** | **P-value** |
| --- | --- | --- | --- | --- | --- | --- | --- |
| **Thickness** | | | | | | | |
| PRP | 7.6 (6.6, 8.6) | 7.5 (6.5, 8.5) | 7.0 (6.0, 8.0) | -0.16 (-0.96, 0.65) | 0.86 | -0.64 (-1.45, 0.16) | .14 |
| NT | 6.2 (5.2, 7.1) | 6.4 (5.4, 7.3) | 6.4 (5.4, 7.4) | 0.20 (-0.60, 1.00) | 0.78 | 0.21 (-0.67, 1.09) | .80 |
| SH | 8.0 (7.0, 9.0) | 8.1 (7.0, 9.1) | 6.7 (5.7, 7.7) | 0.08 (-0.73, 0.89) | 0.95 | -1.30 (-2.11, -0.49) | **.001** |
| *group-level* | NT vs SH **p = .03** |  |  |  |  | PRP vs SH **p = .02** | .**04** |
| **Echotexture** | | | | | | | |
| PRP | 1.9 (1.3, 2.5) | 1.9 (1.3, 2.5) | 1.9 (1.3, 2.5) | 0.0 (-0.64, 0.64) | 1 | -8.1 (-14.6, -1.6) | 1 |
| NT | 2.0 (1.5, 2.5) | 1.8 (1.3, 2.4) | 2.0 (1.4, 2.6) | -0.17 (-0.80, 0.46) | 0.76 | -0.01 (-0.70, 0.68) | .99 |
| SH | 2.0 (1.4, 2.6) | 2.4 (1.9, 3.0) | 1.7 (1.1, 2.3) | 0.44 (-0.20, 1.09) | 0.21 | -0.33 (-0.98, 0.31) | .40 |
| *group-level* | p = .95 |  |  |  |  |  | .193 |
| **Hyperemia** | | | | | | | |
| PRP | 1.8 (1.1, 2.5) | 1.3 (0.7, 2.0) | 1.1 (0.4, 1.8) | -0.44 (-1.12, 0.23) | 0.24 | -0.67 (-1.34, 0.01) | .053 |
| NT | 1.2 (0.6, 1.8) | 1.1 (0.4, 1.7) | 0.5 (-0.2, 1.2) | -0.10 (-0.76, 0.57) | 0.91 | -0.67 (-1.40, 0.06) | .07 |
| SH | 2.1 (1.4, 2.8) | 2.0 (1.3, 2.7) | 1.1 (0.4, 1.8) | -0.11 (-0.79, 0.56) | 0.89 | -1.00 (-1.67, -0.33) | **.003** |
| *group-level* | P = .13 |  |  |  |  |  | .583 |

**Table S2**. Clinical outcome at baseline, week 16, and week 52 across the three treatment groups for only those patients that were unaffected by temporary restriction of team sport activities. Reported as estimated mean (95% CI) from longitudinal data analysis with subject as a random effect. PRP=platelet-rich plasma, NT=needle tenotomy, SH=sham, VISA-P = PT-specific Victorian Institute of Sport Assessment Patella, VAS=visual analogue scale.

|  | **Week 0** | **Week 16** | **Week 52** | **Week 16-0** | **P-value** | **Week 52-0** | **P-value** |
| --- | --- | --- | --- | --- | --- | --- | --- |
| **VAS** (0 - 10) | | | | | | | |
| PRP | 7.3 (5.7, 8.8) | 4.2 (2.6, 5.8) | 1.5 (-0.1, 3.2) | -3.06 (-4.86, -1.26) | < 0.001 | -5.70 (-7.58, -3.82) | **< 0.001** |
| NT | 6.8 (5.4, 8.2) | 5.4 (3.9, 7.0) | 4.9 (3.2, 6.6) | -1.39 (-3.15, 0.36) | 0.15 | -1.86 (-3.80, 0.08) | 0.06 |
| SH | 7.7 (5.8, 9.5) | 4.7 (2.8, 6.5) | 4.8 (3.0, 6.7) | -3.00 (-5.08, -0.92) | 0.003 | -2.83 (-4.91, -0.76) | **0.005** |
| *group-level* | p = .36 |  |  |  |  |  | **0.007** |
| **VISA-P** (0 - 100) | | | | | | | |
| PRP | 46.6 (32.7, 60.5) | 67.4 (53.5, 81.3) | 76.7 (62.9, 90.6) | 20.75 (3.89, 37.61) | 0.012 | 30.12 (13.26, 46.99) | **< 0.001** |
| NT | 58.9 (46.4, 71.4) | 69.5 (55.8, 83.1) | 67.9 (52.8, 83.1) | 10.60 (-5.81, 27.00) | 0.29 | 9.03 (-9.08, 27.15) | 0.48 |
| SH | 55.5 (39.4, 71.6) | 65.2 (49.0, 81.3) | 72.6 (55.4, 89.8) | 9.67 (-9.81, 29.14) | 0.49 | 17.08 (-3.63, 37.80) | 0.13 |
| *group-level* | p = .36 |  |  |  |  |  | 0.25 |
| **Tegner Activity Scale** (1-10) | | | | | | | |
| PRP | 3.9 (2.6, 5.1) | 5.1 (3.8, 6.3) | 5.7 (4.4, 6.9) | 1.19 (-0.18, 2.55) | 0.1 | 1.81 (0.45, 3.18) | **0.006** |
| NT | 5.7 (4.6, 6.8) | 5.4 (4.2, 6.6) | 6.3 (5.0, 7.6) | -0.34 (-1.67, 1.00) | 0.84 | 0.63 (-0.85, 2.10) | 0.6 |
| SH | 6.0 (4.6, 7.4) | 5.7 (4.2, 7.1) | 6.3 (4.9, 7.8) | -0.33 (-1.91, 1.24) | 0.89 | 0.33 (-1.24, 1.91) | 0.89 |
| *group-level* | p = .04; no sig. 2-way comparison after adjustment |  |  |  |  |  | 0.25 |
