# Supplementary material for: Platelet-rich plasma for patellar tendinopathy: a randomized controlled trial correlating clinical outcomes and quantitative imaging
Source: Radiol Adv. 2024 Jul 8;1(2):umae017. doi: 10.1093/radadv/umae017 (PMC12481695; doi:10.1093/radadv/umae017)

***Platelet-rich plasma for patellar tendinopathy: A randomized controlled trial correlating clinical outcomes and quantitative ultrasound and MR imaging***

Manuscript type

Original research

Summary statement

**Platelet-rich plasma injection improved pain over a 1-year period compared to needle tenotomy and sham, wherein the healing response can be assessed with quantitative ultrasound Shear-Wave Elastography** **and Ultrashort-TE** **MRI.**

Key results

- There was clinical improvement at 52-weeks across treatment groups irrespective of intervention, wherein the largest improvement was seen when PRP was applied.
- At 52-weeks, there was a moderate correlation between the change in ultrasound shear wave speeds with improvement in pain across treatment groups (PRP, needle tenotomy, sham).
- At 52-weeks, the fraction of bound water increased significantly across all treatment groups, while a significant decrease in T2*_single_ was only seen with MRI in the patients treated with PRP.

Abbreviations

NT: needle tenotomy

PRP: platelet rich plasma

PT: patellar tendinopathy

RCT: randomized-controlled trial

SH: sham

SWS: shear wave speed

UTE: Ultrashort echo time

VAS: Visual analog scale

VISA-P: Patella tendinopathy-specific Victorian Institute of Sport Assessment Patella

**Abstract**

Background: Patellar tendinopathy (PT) is a common overuse injury in active individuals, often with incomplete recovery. Platelet-rich plasma (PRP) treatment has shown promising results. Advanced quantitative imaging, such as ultrashort echo-time (UTE) MRI and ultrasound (US) shear-wave elastography (SWE) may be valuable adjuncts to traditional qualitative markers as indicators of treatment response.

Purpose: To investigate the clinical outcomes and quantitative imaging changes in adults with symptomatic patellar tendinopathy treated with PRP, needle tenotomy (NT) or sham injection (SH).

Materials and Methods: Single-blinded single center prospective randomized controlled trial from April 2017 until July 2022 with three parallel interventions in athletes with symptomatic PT: PRP, NT or SH. Visual analog scale (VAS) pain, Patella tendinopathy-specific Victorian Institute of Sport Assessment Patella (VISA-P) function, conventional US, shear wave speed (SWS), UTE T2* relaxation time (T2*_single_) and T2* fraction of fast-relaxing macromolecular-bound water (F_F_) were acquired at 0, 16 and 52-weeks. Longitudinal analyses were used to compare intra- and inter-group differences over time, and correlations were assessed.

Results: 29 subjects (mean age, 26.1±5.3 years; 82.8% men) were randomized. At 52-weeks all groups demonstrated a significant improvement in pain, though most pronounced within the PRP group ($\Delta$VAS=-5.9, 95% Confidence Interval [-7.8, -3.9], p<.001). SWS increased significantly only in the PRP group ($\Delta$+2.3, [0.8, 3.9], p=.003). Change in SWS was moderately correlated with change in pain across all groups (r=-.52, [-.76, -.15], p=.009). F_F_ significantly increased in all groups ($\Delta$=0.10-0.11, p=.024-0.046); a significant decrease in T2*_single_ was only seen in the PRP group ($\Delta$=-8.07, [-14.6, -1.55], p=.014).

Conclusion: Clinical improvement was evident irrespective of treatment but was greatest with PRP. SWS correlated with improvement in pain and may reflect an acceptable adjunct to assess healing in patellar tendinopathy. Changes in T2* UTE quantitative markers with treatment indicate potential, but further research is needed to clarify its clinical applicability.

**Introduction**

Patellar tendinopathy (PT) is a common condition primarily affecting active individuals engaged in jumping activities.^1^ PT is characterized by pain at the proximal patellar tendon and reduced knee function.^2^ It is thought to be due to overuse induced degeneration and inflammatory changes in the patellar tendon.^3^ Structural changes include collagen disorganization, increased proteoglycan and glycosaminoglycan (GAG), increased free bulk water and decreased macromolecular bound water and neovascularization.^4–6^ Conservative treatment is not consistently successful which has led to exploration of minimally invasive interventions. Many of these interventions, including platelet-rich plasma injection (PRP) and needle tenotomy (NT), addresses the structural changes through recruitment of growth factors to the site of the injury.^7^

Imaging is important for diagnosing PT, but assessment of treatment response remains challenging. Conventional qualitative imaging techniques provide subjective information which do not always correlate with outcomes.^8^ Quantitative US and MRI imaging techniques, such as shear wave elastography (SWE) and ultrashort echo time (UTE) T2* MRI mapping, have shown potential for objectively assessing tendinopathy. ^9–19^

SWE uses an acoustic radiation force pulse to generate shear waves perpendicular to the ultrasound beam causing transient tissue displacements. The corresponding shear-wave speeds (SWS) are directly related to the shear modulus, an absolute measure of the tissue’s elastic properties, wherein shear waves propagate slower through disorganized tendons than in normal compact fibrillar tendons.^17^ Previous literature has suggested that SWS may be a more objective and sensitive marker for tissue healing than conventional US.^10^

UTE-MRI visualizes tissues with ultrashort TE, such as dense collagen of tendon, by capturing the rapidly decaying signal undetectable with conventional T2 imaging.^20^ UTE-T2* relaxation time can be quantified by applying a single-component or bi-component model. In prior studies, a decrease in single-component T2* relaxation time (T2*_single_) has correlated with improved clinical outcome in PT after treatment.^15^ However, T2*_single_ is a nonspecific parameter, averaging the relaxation characteristics of the individual water components of the tissue. Bi-component UTE-T2* mapping may provide more specific information of both macromolecular water-bound and free water component in tendon healing.^11–16,21^

This single-blinded single center randomized controlled trial (RCT) investigated the relative effectiveness of three parallel interventions: PRP, needle tenotomy (NT) or sham injection (SH) in the treatment of PT at 52-week follow-up as measured by patient reported outcomes (PROs). In addition, this study investigated the role of quantitative imaging parameters—SWS and UTE-T2* single-component and bi-component parameters—as a marker of patellar tendon healing.

**Materials and methods**

Trial design

The study was a single-blinded multi-arm parallel group RCT comprising three interventions for PT (PRP, NT and SH). Subjects were recruited and block randomized by the study nurse using an allocation ratio of 1:1:1. Subjects were blinded to treatment group and blood was drawn in all participants. A musculoskeletal radiologist (KL) with 20 years of experience performed all interventions. PRP included single ultrasound-guided injection of 5 mL autologous leucocyte-rich PRP (Arthrex ACP Double Syringe System, Arthrex Inc., Naples, FL) within the abnormal tendon and the enthesis after 10 needle passes. NT included 20 passes with a 22G, 1.5-inch needle targeting hypoechoic and hyperemic tendon. SH included 20 needle passes only in the subcutaneous tissues superficial to the patella tendon. Two blinded independent researchers (RvdH, ZS) post-processed all data. An independent statistician (SH) performed all statistical analyses.

Participants

IRB and written informed consent were obtained, and HIPAA was adhered to. Patients with PT were diagnosed by a sports medicine specialist (JW) with 15 years of experience at the University Hospital Orthopedics Clinics. Inclusion criteria included: 18-39 years, chronic (>3 months) PT, MRI or US evidence of PT, Visual Analogue Scale (VAS) pain score of >3, self-reported failure of >2 conservative treatments (e.g., NSAIDs, relative rest, ice, and bracing) and supervised physical therapy. Exclusion criteria included: inability to comply with follow-up, hematologic conditions, knee pain from other etiologies (e.g., degenerative joint disease, trauma), full or partial patellar tendon tear, use of anticoagulation or immunosuppression, prior surgery, pregnancy, worker’s compensation injury, daily opioid use, MRI contraindication, systemic diseases such as diabetes or connective tissue diseases and prior PRP or NT procedure.

Clinical and US outcome measures

VAS during activity, PT-specific Victorian Institute of Sport Assessment Patella (VISA-P) score and Tegner Activity Scale were collected at baseline, 16-weeks, and 52-weeks.^22,23^

Both conventional (B-mode and Power-doppler) and quantitative (SWE) US images were obtained by a single operator (KL) at all timepoints using a linear high frequency (6-15 MHz) transducer (Supersonic Imagine, Aix-en-Provence, France) with a gel stand-off pad in long axis to the patella tendon with the knee in 30° flexion as measured by a goniometer.

A sagittal three-dimensional multi-echo UTE-T2* sequence (3D-Cones, GE Healthcare, Waukesha, WI) of the knee was performed at all time points using a 3.0T scanner (Discovery MR750, GE Healthcare, Waukesha, WI) and 8-channel phased-array extremity coil (InVivo, Orlando, FL). The sequence utilized a 3D cone k-space sampling scheme with 16 echo times between 0.003ms and 35ms. Additional parameters included a 40ms repetition time, 20° flip angle, 16cm field-of-view, ±150KHz bandwidth, 256×256 matrix, 3mm slice thickness, one excitation, 10 slices through the patellar tendon, and 19-minute scan time.

Image Post-Processing and Analysis

A musculoskeletal radiologist with 5 years of experience (ZS) retrospectively reviewed all conventional and SWE US images. The thickest portion of the tendon (mm), and hypo-echogenicity and hyperemia [(0=normal) to (3=severe)] were reported. The SWS maps had three 10 mm diameter regions of interest (ROIs) corresponding to diseased areas on the baseline B-mode images (Figure 1). SWS was averaged across the ROIs. Care was taken to measure SWS in the exact same location on follow-up.

For MRI, a musculoskeletal radiologist with 6 years of experience (RvdH) delineated ROIs of the proximal 1 cm of the patellar tendon on TE 4.3ms UTE image using 3D Slicer (Figure 2). All echoes were registered using rigid registration.^24^ Single-component T2*_single_ and bicomponent fast-relaxing macromolecular-bound water component (T2*_fast_), slow relaxing bulk water component (T2*_slow_) and fraction of fast-relaxing macromolecular-bound water component (F_F_) parameter maps were calculated by voxel-by-voxel curve fitting using MATLAB (MATLAB 2010b, MathWorks Inc, Natick, MA).^25^ After superimposing the ROI over the UTE-T2* parameter maps, mean and SD values were obtained. Excellent repeatability has been demonstrated in a prior study.^13^

Statistics

The sample size was calculated for comparing VISA-P score change from baseline to 52-weeks between groups. With an expected Cohen’s D effect size of 0.52, 20 subjects per group was estimated to result in a 95% power. With 29 total subjects, prior to analysis, there would be 65% power to detect an effect size of 0.52 and 80% power to detect a slight larger, but still attainable, effect size of 0.61. All analyses followed intention-to-treat. Missing data were treated as missing at random.

Longitudinal data analysis (LDA) with subject as a random effect were used to compare between group and within group differences in mean outcome measures over time. Group allocation, a 3-level factor for time, and their interaction term were fixed predictors in the LDA models. Pearson’s correlation was used to assess correlations of change from baseline to 52-weeks between outcome measures. To account for multiple comparisons, a Tukey’s family-wise adjustment was done in the case of a post-hoc examination of the 2-way comparison. P-values <0.05 were considered significant. All statistical analyses were performed in R v4.0.

**Results**

Study population

897 patients were screened, and ultimately 29 athletes were enrolled between April 2017 and July 2022 and randomized into the following groups: PRP=9; NT=11; and SH=9 (Figure 3, Table 1). One patient in the NT group did not receive the intervention because the patient did not qualify per imaging criteria for PT. One patient from the NT group was lost to follow-up. For the MRI analyses, one patient in the SH group was excluded due to mid-tendon rather than proximal tendon. Three patients (1x PRP, 2x NT) were excluded from the MRI analysis for varying technical reasons such as failed registration and artifacts. MRI data was available for 19 patients at baseline, 20 patients at 16-weeks, and for 16 patients at 52-weeks. No adverse events were reported.

The patients in the three treatment arms were not statistically different with respect to age, BMI, sex, and race. Most participants were Caucasian. A total of 5 patients were affected by temporary (~5 month) restriction of team sport activities relating to the COVID-19 pandemic.^25^ Of these 5 patients, 3 were in the SH cohort, and there was 1 patient from both the NT and PRP cohorts. All five participants demonstrated a higher-than-average reduction in VAS pain scores, most pronounced in the SH and NT groups.

Outcome

All groups demonstrated an improvement in VAS pain during activity at 16-weeks, which was only statistically significant in the PRP and SH group (Table 2). At 52-weeks all groups demonstrated a significant improvement in pain during activity with this effect appearing most pronounced in the PRP group (Table 2). At 16-weeks, the VISA-P score improved in all groups, but this improvement was only significant for the PRP group (D20.3, 95%CI [5.0, 35.7], p=0.006). At 52 weeks, the VISA-P score improved in all groups, but this improvement was only statistically significant for the PRP and SH groups (Table 2). On the Tegner Activity Scale, only the PRP group demonstrated a significant improvement at 52-weeks (Table 2).

At baseline, the NT group had a significantly smaller tendon thickness (p = .027) and higher SWS (p = .025) than the SH group. The baseline conventional and qualitative US parameters were otherwise not significantly different amongst the treatment groups at baseline. At 16-weeks there was no significant change in the US parameters in any of the treatment groups. At 52-weeks, only the SH group experienced a significant change in the conventional US parameters with a decrease in both tendon thickness and hyperemia. There was no change in the hypo-echogenicity in any of the treatment groups at 52-weeks. There was a significant increase in SWS in only the PRP group at 52-weeks (Table 3, Figure 4). The change in conventional US measures was not significantly correlated with the change in VAS (Table 4). The change in SWS at 52-weeks was moderately correlated with the change in VAS (-0.52 [-0.76, -0.15] p= .009) (Figure 5).

The NT group had a significantly lower T2*_fast_ (p = .027) compared to the SH group at baseline (Table 3). The baseline quantitative MRI parameters were otherwise not significantly different amongst the treatment. At 16-weeks, there was no significant change in the UTE T2* parameters in any of the treatment groups. F_F_ significantly increased from baseline to 52-weeks in all groups (Figure 6). Only the PRP group demonstrated a significant decrease in T2*_Single_ (Figure 6). There was no significant change in T2*_fast_ or T2*_slow_ in any of the groups at 52-weeks. The changes in T2*_single_ and F_F_ at 52-weeks were moderately correlated with the change in VAS, respectively, but the p-values did not show statistical significance for these correlations (Table 4, Figure 5).

No correlations were demonstrated between either UTE measures or SWS and VISA-P. Also, no significant correlation was demonstrated between quantitative MRI parameters and SWS. F_F_ was significantly correlated with a decrease in hyperemia (-0.62 [-0.88, -0.07] p=.030).

**Discussion**

Previous studies have assessed clinical outcomes after treatment of PT with PRP^26^ However, no previous placebo-controlled RCT has compared PRP to NT using multimodal imaging to assess tendon healing over 52-weeks. This study demonstrated improvement in VAS across all treatment groups at 52-weeks; however, only the PRP group demonstrated significant improvement in all PROs. Furthermore, there was a moderate correlation between the change in SWS and VAS at 52-weeks across treatment groups. Only the PRP group showed a significant decrease in T2*_single_ and increase in SWS at 52-weeks, while all groups demonstrated an increase in F_f_ at 52-weeks.

A key finding was the improvement in VAS across all treatment groups, including SH, which suggests improvement without any minimal invasive treatment.^27^ Activity restrictions imposed by the COVID-19 pandemic may have contributed to improvement in pain especially in the sham group, because the lockdown affected a larger proportion of the subjects in this arm. The placebo effect could also have contributed to these results.^28^

Despite improvement in all groups, the PRP cohort reported the greatest improvement in VAS and only the PRP cohort reported significant clinical improvement in PROs at 52-weeks. The role of PRP in PT and how it compares to other interventions is still unclear.^7^ A 2022 meta-analysis found that PRP injections did not provide clinical benefit in terms of pain relief compared to other non-PRP injection alternatives.^29^ Additionally, a 2021 systematic review and meta-analysis concluded that their “most striking finding” was “the absence of high-quality evidence to convincingly demonstrate superior outcomes with specific treatment modalities (for patella tendinopathy)”.^30^

Current conventional qualitative imaging does not always correlate with clinical outcomes. Prior studies have suggested that ultrasound SWE may serve as a quantitative imaging metric to assess tendon healing.^17,18,31^ In this study, increase in SWS was correlated with improvement in PT-related pain, adding further credence to this notion. Interestingly, no significant correlation was found between the change between SWS and the other PROs.

F_f,_ increased significantly across all groups at 52-weeks, while T2*_single_ only decreased significantly in the PRP group. This difference may be because T2*_single_ is less specific than F_f_ while T2*_single_ reflects a composite measure of the T2* relaxation characteristics of all water components rather than assessing the individual macromolecular and bulk water components. Alternatively, these results may have simply been affected by a lack of power. The increase in Ff at 52-weeks was likely due to an increase in macromolecular bound water and decrease in bulk water due to collagen fiber proliferation and remodeling during tendon healing.^32^ There were moderate correlations with T2*_single_ and F_f_, respectively, with VAS at 52-weeks—albeit with p-values above the threshold for statistical significance. Other studies have reported correlations with PROs and UTE-MRI.^12^ It is possible that the results in this study may have been affected by the small sample size.

There was no correlation between SWS and F_f,_ or T2*_single_. This may be due to a lack of power or due to a true difference between imaging parameters. SWS reflects tendon elastic properties which is primarily dependent upon collagen fiber organization and macrostructure, while UTE-MRI may be more reflective of tendon biochemical composition and microstructure.^12,33^ This may explain the observed correlation of US hyperemia only with F_F_. This suggests that quantitative US and MR imaging may serve as complementary, rather than interchangeable, modalities.

This study has several limitations. The COVID-19 pandemic limited recruitment and may have affected the participants’ activity level, indirectly affecting their clinical outcomes, especially in the sham group. The study was also limited by the small sample size and inability to acquire a substantial number of UTE-MRI follow-up data points. This precluded statistical adjustments for confounders and the powering necessary to find statistical significance for the correlations between the UTE-MRI parameters and PROs. The missing UTE-MRI data was equally distributed across groups, and the missing data points were utilized rather than imputing a large percentage of the data. A single operator performed all SWS US measurements. This was by design, as previous studies have shown good intra-rater reliability while inter-rater reliability is less reliable.^34,35^ This design, however, did limit the ability to perform any inter-rater reliability measures for SWS. Finally, the study was limited by demographic homogeneity.

In summary, this study suggests that improvement in pain may be the natural course of PT with rest and physical therapy. However, the greatest degree of clinical improvement was with PRP treatment when compared to SH and NT. Additionally, this study demonstrated that quantitative imaging may have a potential role as an adjunct measure of tendon healing. The promising results of this study may help form the basis for future larger and more diverse clinical trials necessary to elucidate the true clinical role of emerging minimally invasive treatments and quantitative imaging tools.

**References**

1. Ferretti A, Ippolito E, Mariani P, Puddu G. Jumper’s Knee. *Am J Sports Med*. 1983;11(2):58-62. doi:10.1177/036354658301100202

2. Zwerver J, Mc Auliffe S, Rio EK, Scott A, Vicenzino BT, Weir A. ICON 2019—international scientific tendinopathy symposium: building an ICONic tendon tower—launching a new era in clinical tendinopathy research. *Br J Sports Med*. 2020;54(8):442-443. doi:10.1136/bjsports-2019-101214

3. Fredberg U, Stengaard‐Pedersen K. Chronic tendinopathy tissue pathology, pain mechanisms, and etiology with a special focus on inflammation. *Scand J Med Sci Sports*. 2008;18(1):3-15. doi:10.1111/j.1600-0838.2007.00746.x

4. de Mos M, van El B, DeGroot J, et al. Achilles Tendinosis. *Am J Sports Med*. 2007;35(9):1549-1556. doi:10.1177/0363546507301885

5. Xu Y, Murrell GAC. The Basic Science of Tendinopathy. *Clin Orthop Relat Res*. 2008;466(7):1528-1538. doi:10.1007/s11999-008-0286-4

6. Riley G. The pathogenesis of tendinopathy. A molecular perspective. *Rheumatology*. 2004;43(2):131-142. doi:10.1093/rheumatology/keg448

7. Stewart ZE, Lee K. Lower extremity ultrasound-guided interventions: tendon, ligament, and plantar fascia. *Skeletal Radiol*. 2023;52(5):991-1003. doi:10.1007/s00256-022-04212-4

8. Docking SI, Ooi CC, Connell D. Tendinopathy: Is Imaging Telling Us the Entire Story? *Journal of Orthopaedic & Sports Physical Therapy*. 2015;45(11):842-852. doi:10.2519/jospt.2015.5880

9. Crawford SK, Thelen D, Yakey JM, Heiderscheit BC, Wilson JJ, Lee KS. Regional shear wave elastography of Achilles tendinopathy in symptomatic versus contralateral Achilles tendons. *Eur Radiol*. 2022;33(1):720-729. doi:10.1007/s00330-022-08957-3

10. Dirrichs T, Quack V, Gatz M, et al. Shear Wave Elastography (SWE) for Monitoring of Treatment of Tendinopathies. *Acad Radiol*. 2018;25(3):265-272. doi:10.1016/j.acra.2017.09.011

11. Liu J, Nazaran A, Ma Y, et al. Single- and Bicomponent Analyses of T2 <math id="M1"> <mrow> <mo>⁎</mo> </mrow> </math> Relaxation in Knee Tendon and Ligament by Using 3D Ultrashort Echo Time Cones (UTE Cones) Magnetic Resonance Imaging. *Biomed Res Int*. 2019;2019:1-9. doi:10.1155/2019/8597423

12. Loegering IF, Denning SC, Johnson KM, Liu F, Lee KS, Thelen DG. Ultrashort echo time (UTE) imaging reveals a shift in bound water that is sensitive to sub-clinical tendinopathy in older adults. *Skeletal Radiol*. 2021;50(1):107-113. doi:10.1007/s00256-020-03538-1

13. Kijowski R, Wilson JJ, Liu F. Bicomponent ultrashort echo time analysis for assessment of patients with patellar tendinopathy. *Journal of Magnetic Resonance Imaging*. 2017;46(5):1441-1447. doi:10.1002/jmri.25689

14. Juras V, Apprich S, Szomolanyi P, Bieri O, Deligianni X, Trattnig S. Bi-exponential T2* analysis of healthy and diseased Achilles tendons: an in vivo preliminary magnetic resonance study and correlation with clinical score. *Eur Radiol*. 2013;23(10):2814-2822. doi:10.1007/s00330-013-2897-8

15. Breda SJ, de Vos R, Poot DHJ, Krestin GP, Hernandez‐Tamames JA, Oei EHG. Association Between T _2_ ^*^ Relaxation Times Derived From Ultrashort Echo Time <scp>MRI</scp> and Symptoms During Exercise Therapy for Patellar Tendinopathy: A Large Prospective Study. *Journal of Magnetic Resonance Imaging*. 2021;54(5):1596-1605. doi:10.1002/jmri.27751

16. Pauli C, Bae WC, Lee M, et al. Ultrashort–Echo Time MR Imaging of the Patella with Bicomponent Analysis: Correlation with Histopathologic and Polarized Light Microscopic Findings. *Radiology*. 2012;264(2):484-493. doi:10.1148/radiol.12111883

17. Quilling GM, Lee KS, Ebben B. Shear wave elastography imaging in a porcine tendinopathy model. *Skeletal Radiol*. 2022;51(11):2167-2173. doi:10.1007/s00256-022-04073-x

18. Aubry S, Risson JR, Kastler A, et al. Biomechanical properties of the calcaneal tendon in vivo assessed by transient shear wave elastography. *Skeletal Radiol*. 2013;42(8):1143-1150. doi:10.1007/s00256-013-1649-9

19. Lee KS, Martin J, Thelen D. Science to Practice: Quantitative US Elastography Can Be Used to Quantify Mechanical and Histologic Tendon Healing in a Rabbit Model of Achilles Tendon Transection. *Radiology*. 2017;283(2):311-313. doi:10.1148/radiol.2017170126

20. Chang EY, Du J, Chung CB. UTE imaging in the musculoskeletal system. *Journal of Magnetic Resonance Imaging*. 2015;41(4):870-883. doi:10.1002/jmri.24713

21. Liu F, Kijowski R. Assessment of different fitting methods for in-vivo bi-component T2* analysis of human patellar tendon in magnetic resonance imaging. *Muscle, Ligaments and Tendons Journal*. 2017;7(1):163. doi:10.11138/mltj/2017.7.1.163

22. Visentini PJ, Khan KM, Cook JL, Kiss ZS, Harcourt PR, Wark JD. The VISA score: An index of severity of symptoms in patients with jumper’s knee (patellar tendinosis). *J Sci Med Sport*. 1998;1(1):22-28. doi:10.1016/S1440-2440(98)80005-4

23. Tegner Y, Lysholm J. Rating Systems in the Evaluation of Knee Ligament Injuries. *Clin Orthop Relat Res*. 1985;198:42-49.

24. Klein S, Staring M, Murphy K, Viergever MA, Pluim J. elastix: A Toolbox for Intensity-Based Medical Image Registration. *IEEE Trans Med Imaging*. 2010;29(1):196-205. doi:10.1109/TMI.2009.2035616

25. Sabbagh RS, Shah NS, Kanhere AP, Hoge CG, Thomson CG, Grawe BM. Effect of the COVID-19 Pandemic on Sports-Related Injuries Evaluated in US Emergency Departments. *Orthop J Sports Med*. 2022;10(2):232596712210753. doi:10.1177/23259671221075373

26. Dragoo JL, Wasterlain AS, Braun HJ, Nead KT. Platelet-Rich Plasma as a Treatment for Patellar Tendinopathy. *Am J Sports Med*. 2014;42(3):610-618. doi:10.1177/0363546513518416

27. Rosen AB, Wellsandt E, Nicola M, Tao MA. Clinical Management of Patellar Tendinopathy. *J Athl Train*. 2022;57(7):621-631. doi:10.4085/1062-6050-0049.21

28. Miller FG, Colloca L, Kaptchuk TJ. The Placebo Effect: Illness and Interpersonal Healing. *Perspect Biol Med*. 2009;52(4):518-539. doi:10.1353/pbm.0.0115

29. Barman A, Sinha MK, Sahoo J, et al. Platelet-rich plasma injection in the treatment of patellar tendinopathy: a systematic review and meta-analysis. *Knee Surg Relat Res*. 2022;34(1):22. doi:10.1186/s43019-022-00151-5

30. Challoumas D, Pedret C, Biddle M, et al. Management of patellar tendinopathy: a systematic review and network meta-analysis of randomised studies. *BMJ Open Sport Exerc Med*. 2021;7(4):e001110. doi:10.1136/bmjsem-2021-001110

31. DeWall RJ, Slane LC, Lee KS, Thelen DG. Spatial variations in Achilles tendon shear wave speed. *J Biomech*. 2014;47(11):2685-2692. doi:10.1016/j.jbiomech.2014.05.008

32. Thomopoulos S, Parks WC, Rifkin DB, Derwin KA. Mechanisms of tendon injury and repair. *Journal of Orthopaedic Research*. 2015;33(6):832-839. doi:10.1002/jor.22806

33. Kubo K, Kawakami Y, Fukunaga T. Influence of elastic properties of tendon structures on jump performance in humans. *J Appl Physiol*. 1999;87(6):2090-2096. doi:10.1152/jappl.1999.87.6.2090

34. Ito N, Sigurðsson HB, Pohlig RT, Cortes DH, Grävare Silbernagel K, Sprague AL. Reliability of Continuous Shear Wave Elastography in the Pathological Patellar Tendon. *Journal of Ultrasound in Medicine*. 2023;42(5):1047-1055. doi:10.1002/jum.16115

35. Taş S, Onur MR, Yılmaz S, Soylu AR, Korkusuz F. Shear Wave Elastography Is a Reliable and Repeatable Method for Measuring the Elastic Modulus of the Rectus Femoris Muscle and Patellar Tendon. *Journal of Ultrasound in Medicine*. 2017;36(3):565-570. doi:10.7863/ultra.16.03032

**Tables**

**Table 1 Patient characteristics**

|  | **Total** | **Platelet rich plasma** | **Needle tenotomy** | **Sham** | **p-value** |
| --- | --- | --- | --- | --- | --- |
| **N** | 29 | 9 | 11 | 9 |  |
| **Age** *mean (sd)* | 26.1 (5.3) | 25.7 (4.9) | 26.6 (5.6) | 25.8 (5.9) | 0.908 |
| **BMI** *mean (sd)* | 26.4 (5.0) | 26.9 (5.8) | 25.2 (2.9) | 27.4 (6.3) | 0.604 |
| **Sex (M: F)** | 24 (83%) : 5 (17%) | 8 (89%) :1 (11%) | 9 (82%): 2 (18%) | 7 (78%): 2 (22%) | 1 |
| **Race (self-reported)** |  |  |  |  |  |
| African-American (black) | 1 | 1 (11.1%) | 0 (0.0%) | 0 (0.0%) |  |
| American Indian or Alaska  Native | 1 | 0 (0.0%) | 0 (0.0%) | 1 (11.1%) |  |
| Caucasian | 27 | 8 (88.9%) | 11 (100.0%) | 8 (88.9%) |  |

**Table 2 Clinical outcome for baseline and 52-weeks across the three treatment groups.**

|  | **Week 0** | **Week 16** | **Week 52** | **Week 16-0** | **P-value** | **Week 52-0** | **P-value** |
| --- | --- | --- | --- | --- | --- | --- | --- |
| **VAS** (0 - 10) | | | | | | | |
| PRP | 7.4 (5.9, 9.0) | 4.1 (2.5, 5.6) | 1.6 (0.0, 3.2) | -3.39 (-5.29, -1.49) | < 0.001 | -5.87 (-7.84, -3.89) | <.001 |
| NT | 6.9 (5.5, 8.3) | 5.7 (4.2, 7.2) | 4.5 (2.8, 6.1) | -1.24 (-3.09, 0.62) | 0.26 | -2.45 (-4.47, -0.43) | .01 |
| SH | 7.7 (6.1, 9.2) | 4.4 (2.9, 6.0) | 3.3 (1.8, 4.9) | -3.22 (-5.13, -1.32) | < 0.001 | -4.33 (-6.24, -2.43) | <.001 |
| *group-level* | p = .39 |  |  |  |  |  | .04 |
| **VISA-P** (0 - 100) | | | | | | | |
| PRP | 45.0 (32.2, 57.8) | 65.3 (52.5, 78.1) | 76.8 (64.0, 89.6) | 20.3 (5.0, 35.7) | 0.006 | 31.8 (16.4, 47.1) | <.001 |
| NT | 57.9 (46.3, 69.5) | 69.4 (56.9, 82.0) | 69.3 (55.6, 83.0) | 11.5 (-3.46, 26.5) | 0.17 | 11.4 (-4.9, 27.7) | .23 |
| SH | 56.0 (43.2, 68.8) | 66.6 (53.7, 79.4) | 73.7 (60.3, 87.0) | 10.6 (-4.78, 25.9) | 0.24 | 17.7 (1.7, 33.6) | .03 |
| *group-level* | p = .23 |  |  |  |  |  | .15 |
| **Tegner Activity Scale (1**-10) | | | | | | | |
| PRP | 4.0 (2.8, 5.2) | 4.7 (3.5, 6.0) | 6.1 (4.8, 7.3) | 0.72 (-0.71, 2.15) | 0.478 | 2.06 (0.63, 3.48) | .003 |
| NT | 6.1 (5.0, 7.2) | 5.7 (4.5, 6.9) | 6.6 (5.2, 7.9) | -0.38 (-1.77, 1.02) | 0.82 | 0.47 (-1.06, 1.99) | .78 |
| SH | 6.6 (5.3, 7.8) | 5.9 (4.6, 7.1) | 6.4 (5.2, 7.7) | -0.67 (-2.10, 0.76) | 0.54 | -0.11 (-1.54, 1.32) | .99 |
| *group-level* | PRP vs NT p= .04; PRP vs SH p= .02 |  |  |  |  |  | .32 |

Reported as estimated mean (95% CI) from longitudinal data analysis with subject as a random effect. PRP=platelet rich plasma, NT=needle tenotomy, SH=sham, VISA-P = PT-specific Victorian Institute of Sport Assessment Patella, VAS=Visual analogue scale.

**Table 3 Mean quantitative imaging values (MRI UTE T2*and ultrasound) at baseline to 52-weeks across the three treatment groups.**

|  | **Week 0** | **Week 16** | **Week 52** | **Week 16-0** | **P-value** | **Δ Week 52-0** | **P-value** |
| --- | --- | --- | --- | --- | --- | --- | --- |
| **MRI T2*_single_ (ms)** | | | | | | | |
| PRP | 15.2 (11.8, 18.6) | 11.7 (7.7, 15.7) | 7.1 (2.3, 12.0) | -3.5 (-9.2, 2.2) | .28 | -8.1 (-14.6, -1.6) | .010 |
| NT | 10.6 (6.3, 15.0) | 8.7 (5.0, 12.4) | 6.9 (2.9, 10.9) | -1.9 (-8.2, 4.3) | .68 | -3.7 (-10.2, 2.8) | .32 |
| SH | 14.9 (11.0, 18.9) | 13.4 (9.7, 17.1) | 11.7 (7.7, 15.7) | -1.6 (-7.5, 4.3) | .75 | -3.2 (-9.3, 2.9) | .37 |
| *group-level* | p = .47 |  |  |  |  |  | .75 |
| **MRI F_F_ (%)** | | | | | | | |
| PRP | 0.5 (0.5, 0.6) | 0.6 (0.5, 0.6) | 0.6 (0.5, 0.7) | 0.02 (-0.06, 0.11) | .74 | 0.10 (0.00, 0.20) | .046 |
| NT | 0.6 (0.5, 0.6) | 0.6 (0.5, 0.7) | 0.7 (0.6, 0.7) | 0.03 (-0.07, 0.12) | .75 | 0.11 (0.01, 0.21) | .030 |
| SH | 0.4 (0.4, 0.5) | 0.5 (0.4, 0.6) | 0.5 (0.5, 0.6) | 0.07 (-0.02, 0.16) | .17 | 0.10 (0.01, 0.20) | .020 |
| *group-level* | p = .13 |  |  |  |  |  | .89 |
| **MRI T2*_fast_ (ms)** | | | | | | | |
| PRP | 2.2 (1.7, 2.7) | 1.9 (1.3, 2.5) | 2.1 (1.4, 2.8) | -0.26 (-1.02, 0.50) | .64 | -0.06 (-0.95, 0.82) | .97 |
| NT | 1.8 (1.1, 2.4) | 2.1 (1.5, 2.7) | 2.6 (2.0, 3.2) | 0.33 (-0.52, 1.18) | .57 | 0.82 (-0.07, 1.71) | .07 |
| SH | 2.9 (2.3, 3.5) | 2.6 (2.0, 3.2) | 2.5 (2.0, 3.1) | -0.27 (-1.06, 0.53) | .65 | -0.32 (-1.14, 0.49) | .55 |
| *group-level* | NT vs SH p=.03 |  |  |  |  |  | .27 |
| **MRI T2*_slow_ (ms)** | | | | | | | |
| PRP | 30.0 (24.6, 35.3) | 27.6 (21.4, 33.7) | 28.6 (21.2, 36.1) | -2.4 (-10.6, 5.8) | .71 | -1.3 (-10.8, 8.2) | .91 |
| NT | 27.9 (21.2, 34.6) | 28.8 (23.0, 34.5) | 33.2 (27.1, 39.4) | 0.8 (-8.3, 9.9) | .96 | 5.3 (-4.2, 14.9) | .34 |
| SH | 28.4 (22.2, 34.6) | 29.2 (23.5, 35.0) | 28.6 (22.5, 34.8) | 0.8 (-7.7, 9.4) | .95 | 0.2 (-8.6, 9.0) | .96 |
| *group-level* | p = .92 |  |  |  |  |  | .73 |
| **US SWS (m/s)** | | | | | | | |
| PRP | 6.3 (5.1, 7.4) | 7.3 (6.1, 8.4) | 8.6 (7.4, 9.7) | 1.0 (-0.56, 2.56) | .26 | 2.31 (0.75, 3.88) | .003 |
| NT | 7.6 (6.5, 8.6) | 7.5 (6.3, 8.6) | 7.0 (5.7, 8.3) | -0.09 (-1.60, 1.42) | .98 | -0.60 (-2.24, 1.05) | .62 |
| SH | 5.7 (4.6, 6.9) | 5.9 (4.7, 7.0) | 6.4 (5.2, 7.6) | 0.12 (-1.44, 1.69) | .97 | 0.67 (-0.90, 2.23) | .52 |
| *group-level* | p = .64 |  |  |  |  |  | .78 |

Reported as estimated mean (95% CI) from longitudinal data analysis with subject as a random effect. PRP=platelet rich plasma, NT=needle tenotomy, SH=sham. T2*single =single-component UTE-T2* relaxation time, F_F_ =fraction of the fast-relaxing macromolecular bound water component, T2*fast =fast-relaxing macromolecular-bound water component, T2*slow= slow relaxing bulk water component, SWS=shear wave speed

| **Table 4 Linear correlation of change from baseline to 52-weeks for MRI UTE T2* variables and ultrasound SWS with conventional imaging features and patient reported outcomes.**   \|  \| **Variable** \| **Correlation (95% CI)** \| **P value** \| \| --- \| --- \| --- \| --- \| \| **MRI T2* single** \| \| \| \| \|  \| Thickness \| 0.08 (-0.52, 0.62) \| .81 \| \|  \| Echotexture \| -0.17 (-0.68, 0.45) \| .61 \| \|  \| Hyperemia \| 0.06 (-0.53, 0.62) \| .84 \| \|  \| SWS \| -0.39 (-0.79, 0.24) \| .22 \| \|  \| VAS Pain \| 0.53 (-0.11, 0.86) \| .10 \| \|  \| VISA-P \| 0.07 (-0.55, 0.64) \| .83 \| \| **MRI Ff** \| \| \| \| \|  \| Thickness \| -0.19 (-0.69, 0.43) \| .56 \| \|  \| Echotexture \| -0.30 (-0.75, 0.33) \| .34 \| \|  \| Hyperemia \| **-0.62 (-0.88, -0.07)** \| **.030** \| \|  \| SWS \| 0.03 (-0.55, 0.60) \| .92 \| \|  \| VAS Pain \| -0.51 (-0.85, 0.13) \| .11 \| \|  \| VISA-P \| 0.04 (-0.58, 0.62) \| .91 \| \| **MRI T2* fast** \| \| \| \| \|  \| Thickness \| 0.19 (-0.43, 0.69) \| .56 \| \|  \| Echotexture \| -0.44 (-0.81, 0.18) \| .15 \| \|  \| Hyperemia \| -0.18 (-0.68, 0.44) \| .58 \| \|  \| SWS \| -0.54 (-0.85, 0.04) \| .07 \| \|  \| VAS Pain \| 0.44 (-0.21, 0.82) \| .17 \| \|  \| VISA-P \| 0.18 (-0.47, 0.70) \| .60 \| \| **MRI T2* slow** \| \| \| \| \|  \| Thickness \| -0.03 (-0.59, 0.55) \| .93 \| \|  \| Echotexture \| -0.46 (-0.82, 0.16) \| .13 \| \|  \| Hyperemia \| -0.46 (-0.82, 0.15) \| .13 \| \|  \| SWS \| -0.36 (-0.77, 0.27) \| .26 \| \|  \| VAS Pain \| 0.29 (-0.38, 0.76) \| .40 \| \|  \| VISA-P \| 0.20 (-0.46, 0.71) \| .56 \| \| **US SWS** \| \| \| \| \|  \| Thickness \| -0.39 (-0.68, 0.00) \| .053 \| \|  \| Echotexture \| -0.13 (-0.50, 0.28) \| .52 \| \|  \| Hyperemia \| -0.03 (-0.42, 0.37) \| .88 \| \|  \| VAS Pain \| **-0.52 (-0.76, -0.15)** \| **.009** \| \|  \| VISA-P \| 0.09 (-0.32, 0.48) \| .67 \| |  |
| --- | --- | --- | --- | --- | --- | --- | --- | --- | --- | --- | --- | --- | --- | --- | --- | --- | --- | --- | --- | --- | --- | --- | --- | --- | --- | --- | --- | --- | --- | --- | --- | --- | --- | --- | --- | --- | --- | --- | --- | --- | --- | --- | --- | --- | --- | --- | --- | --- | --- | --- | --- | --- | --- | --- | --- | --- | --- | --- | --- | --- | --- | --- | --- | --- | --- | --- | --- | --- | --- | --- | --- | --- | --- | --- | --- | --- | --- | --- | --- | --- | --- | --- | --- | --- | --- | --- | --- | --- | --- | --- | --- | --- | --- | --- | --- | --- | --- | --- | --- | --- | --- | --- | --- | --- | --- | --- | --- | --- | --- | --- | --- | --- | --- | --- | --- | --- | --- | --- | --- | --- | --- | --- | --- | --- | --- | --- | --- | --- | --- | --- | --- | --- | --- | --- | --- | --- | --- | --- | --- | --- | --- |
| Reported as Pearson correlation coefficient and 95% CI for change from baseline at Week 52, SWS = Shear wave speed. F_F_ = fraction of the fast-relaxing macromolecular bound water component |  |

**Figures and figure legends**

Figure 1. Example of the delineated region of interest (ROI) of the proximal patellar tendon on grey scale and corresponding ultrasound SWS color-map images.


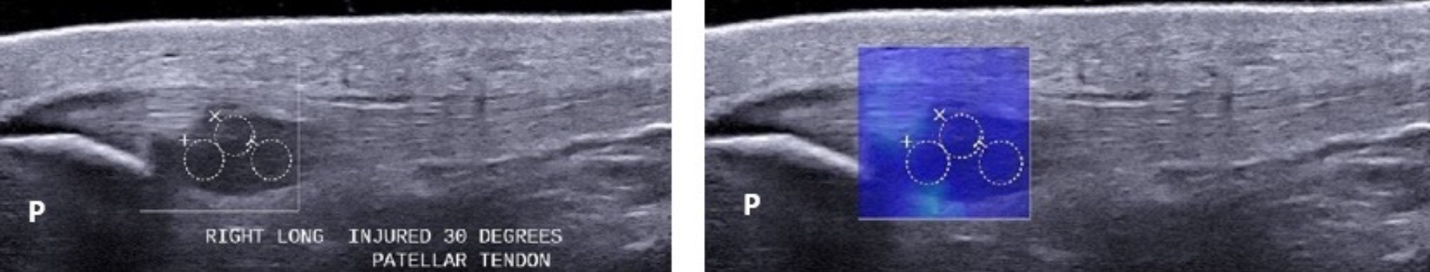


Figure 2. Example of the delineated region of interest (ROI) of the proximal patellar tendon on a MRI UTE-T2* image.


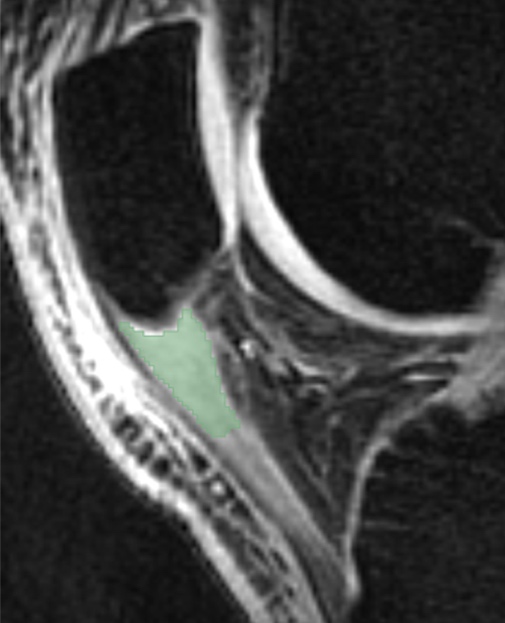


Figure 3. Flowchart of patient inclusion.

Figure 4. Sagittal ultrasound SWS maps at baseline (left) and 52-week follow-up (right) in three patients with patellar tendinopathy (20F, 25M, 26M) following sham, needle tenotomy and PRP treatment respectively. After platelet rich plasma (PRP) treatment statistically significant improvement in SWS from 6.3 to 10.8m/s is demonstrated, while the needle tenotomy and sham treatment showed no improvement over time.


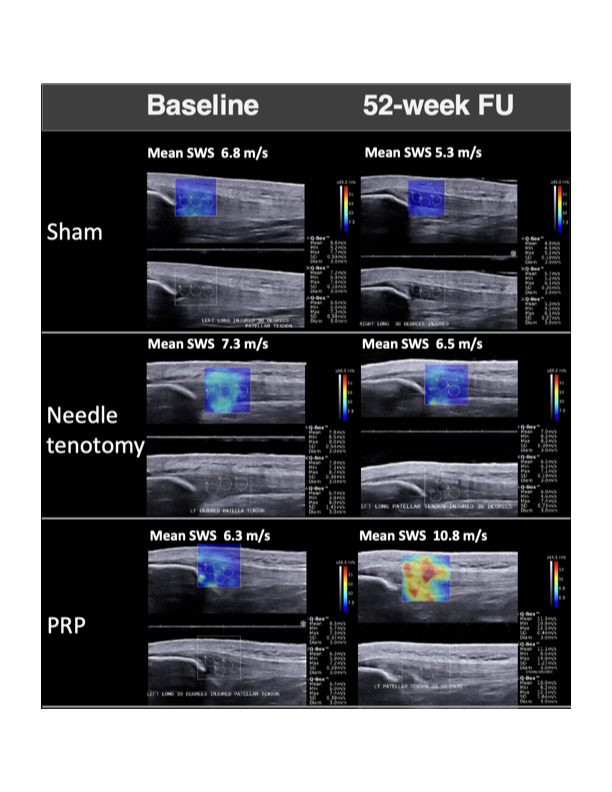


Figure 5. Plots showing correlations between change in VAS pain score and changes in MRI T2*_single,_ MRI F_F_ and ultrasound SWS, respectively, at 52-weeks.

Figure 6. MRI T2*_single_ (ms) and F_F_ (%) parameter maps at baseline and 52-week follow-up in three patients with patellar tendinopathy (20M, 34M, 30M) following sham, needle tenotomy and platelet rich plasma (PRP) treatment respectively. Improvement in T2*_single_ and F_F_ values seen in all groups, but the greatest improvement is demonstrated in the PRP group.


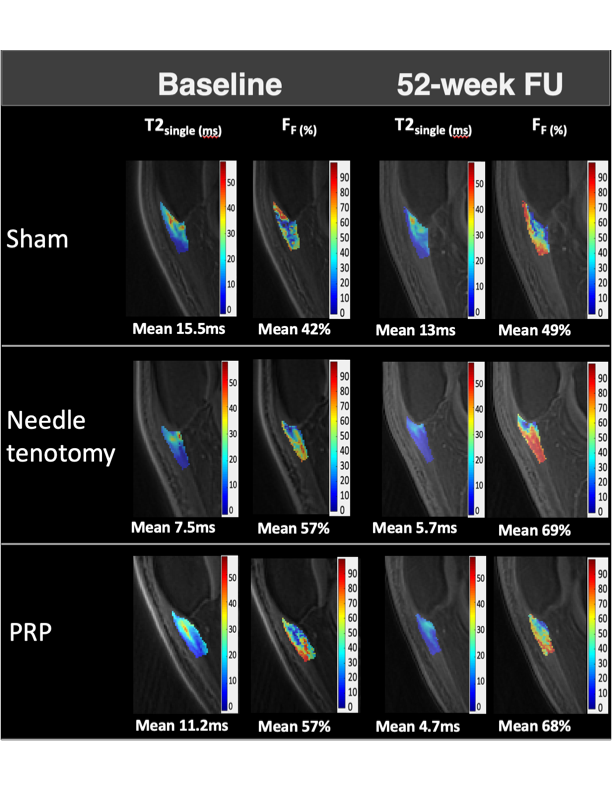

Supplement: umae017_Supplementary_Data [file umae017_Supplementary_Data.zip › text line edited NL SIL 3-18-24.docx]
