# Supplementary material for: Platelet-rich plasma for patellar tendinopathy: a randomized controlled trial correlating clinical outcomes and quantitative imaging
Source: Radiol Adv. 2024 Jul 8;1(2):umae017. doi: 10.1093/radadv/umae017 (PMC12481695; doi:10.1093/radadv/umae017)
Supplement: umae017_Supplementary_Data [file umae017_Supplementary_Data.zip › Manuscript_PL SIL_5-26-24.docx]

***Platelet-Rich Plasma for Patellar Tendinopathy: A randomized controlled trial correlating clinical outcomes and quantitative imaging***

Manuscript type

Original research

Summary statement

**Platelet-rich plasma injection improved pain in patellar tendinopathy over a 1-year period compared to needle tenotomy and sham with treatment response also assessed with quantitative ultrasound and MRI.**

Key results

- There was clinical improvement at 52-weeks across treatment groups irrespective of intervention, with the largest improvement when platelet-rich plasma (PRP) was applied.
- At 52-weeks, there was a moderate correlation between the change in ultrasound shear wave elastography speeds and pain improvement across all treatment groups.
- At 52-weeks, the fraction of bound water seen on ultrashort TE MRI increased significantly across all treatment groups, while a significant decrease in T2*_single_ was only seen with PRP treatment.

Abbreviations

NT: needle tenotomy

PRP: platelet rich plasma

PT: patellar tendinopathy

RCT: randomized-controlled trial

SH: sham

SWE: shear-wave elastography

SWS: shear wave speed

UTE: Ultrashort echo time

VAS: Visual analog scale

VISA-P: Patella tendinopathy-specific Victorian Institute of Sport Assessment Patella

**Abstract**

Background: Patellar tendinopathy (PT) is a common overuse injury in active individuals, often with incomplete recovery. Recently, platelet-rich plasma (PRP) treatment has shown promising results. Traditional qualitative markers are not reliable indicators of treatment response. Advanced quantitative imaging, such as Ultrashort-TE (UTE) MRI and ultrasound (US) shear-wave elastography (SWE) may be valuable adjuncts.

Purpose: To investigate the clinical outcomes and quantitative imaging changes in adults with symptomatic patellar tendinopathy treated with PRP, needle tenotomy (NT) or sham injection (SH).

Materials and Methods: Single-blinded prospective randomized controlled trial from April 2017 until July 2022 with three parallel interventions in athletes with symptomatic PT: PRP, NT and SH. VAS pain, VISA-P function, conventional US, shear wave speed (SWS), UTE T2* relaxation time (T2*_single_) and T2* fraction of fast-relaxing macromolecular-bound water (F_F_) were acquired at 0, 16 and 52-weeks. Longitudinal analyses were used to compare intra- and inter-group differences over time. Correlations were assessed by Pearson’s correlation coefficient.

Results: 29 subjects (mean age, 26.1±5.3 years; 82.8% men) were randomized. At 52-weeks all groups demonstrated a significant improvement in pain, though most pronounced within the PRP group ($\Delta$VAS=-5.9, 95% confidence interval (CI) [-7.8, -3.9], p<.001). SWS increased significantly only in the PRP group ($\Delta$+2.3, [0.8, 3.9], p=.003). Change in SWS was moderately correlated with change in pain across all groups (r=-.52, [-.76, -.15], p=.009). F_F_ significantly increased in all groups ($\Delta$=0.10-0.11, p=.024-0.046); a significant decrease in T2*_single_ was only seen in the PRP group ($\Delta$=-8.07, [-14.6, -1.55], p=.014).

Conclusion: Clinical improvement was evident irrespective of treatment but was greatest with PRP. SWS correlated with improvement in pain and may represent an adjunctive measure to assess healing in patellar tendinopathy. Correlative changes in T2* UTE quantitative markers suggest their potential for response assessment, but further research is needed to clarify their clinical applicability.

**Introduction**

Patellar tendinopathy (PT) is a common condition primarily affecting active individuals engaged in jumping activities.^1^ PT is characterized by pain at the proximal patellar tendon and reduced knee function.^2^ Presumed etiology is overuse induced degeneration and inflammatory changes in the patellar tendon.^3^ Structural changes include collagen disorganization, increased proteoglycan and glycosaminoglycan (GAG), increased free bulk water and decreased macromolecular bound water and neovascularization.^4–6^ Conservative treatment is not consistently successful which has led to exploration of minimally invasive interventions. Many of these interventions, including platelet-rich plasma injection (PRP) and needle tenotomy (NT), address the structural changes through recruitment of growth factors to the site of the injury.^7^

Imaging is important for diagnosing PT, but assessment of treatment response remains challenging. Conventional qualitative imaging techniques provide subjective information but do not always correlate with outcomes.^8^ Quantitative US and MRI techniques, such as shear wave elastography (SWE) and ultrashort echo time (UTE) T2* MRI, have shown potential for objectively assessing tendinopathy.^9–19^

SWE uses an acoustic radiation force pulse to generate shear waves perpendicular to the ultrasound beam causing transient tissue displacements. The corresponding shear-wave velocities (SWS) are directly related to the shear modulus, an absolute measure of the tissue’s elastic properties. SWS are higher in disorganized tendons than in normal compact fibrillar ones.^17^ Previous literature has suggested that SWS may be a more objective and sensitive marker for tissue healing than conventional US.^10^

UTE-MRI visualizes tissues by capturing the rapidly decaying signal undetectable with conventional T2 imaging with ultrashort echo time.^20^ UTE-T2* relaxation time can be quantified by applying a single-component or a bi-component model. In prior studies, decreases in single-component T2* relaxation time (T2*_single_) have been correlated with improved clinical outcome in PT after treatment.^15^ However, T2*_single_ is a nonspecific parameter, averaging the relaxation characteristics of the individual water components of the tissue. Bi-component UTE-T2* mapping may provide more specific information of both macromolecular water-bound and free water components in tendon healing.^11–16,21^

This single-blinded randomized controlled trial (RCT) investigated the relative effectiveness of three parallel PT interventions, PRP, NT, or sham injection (SH), as measured by patient reported outcomes (PROs) with a 52-week follow-up. In addition, this study investigated the role of quantitative imaging parameters—ultrasound SWS and MRI UTE-T2* single-component and bi-component parameters—as a marker of patellar tendon healing.

**Materials and methods**

Trial design

The study was a single-blinded multi-arm parallel group RCT comprising three interventions for PT (PRP, NT and SH). Subjects were recruited and block randomized by the study nurse using an allocation ratio of 1:1:1. Subjects were blinded to treatment group and blood was drawn in all participants. A musculoskeletal radiologist (KL) with 20 years of experience performed all interventions. PRP included single ultrasound-guided injection of 5 mL autologous leucocyte-rich PRP (Arthrex ACP Double Syringe System, Arthrex Inc., Naples, FL) within the abnormal tendon and the enthesis after 10 needle passes. NT included 20 passes with a 22G, 1.5-inch needle targeting hypoechoic and hyperemic tendon. SH included 20 needle passes only in the subcutaneous tissues superficial to the patella tendon. Two blinded independent researchers (RvdH, ZS) post-processed all data. An independent statistician (SH) performed all statistical analyses.

Participants

IRB and written informed consent were obtained, and HIPAA was adhered to. Patients with PT were diagnosed by a sports medicine specialist (JW) with 15 years of experience at the University Hospital Orthopedics Clinics. Inclusion criteria included: 18-39 years, chronic (>3 months) PT, MRI or US evidence of PT, Visual Analogue Scale (VAS) pain score of >3, self-reported failure of >2 conservative treatments (e.g., NSAIDs, relative rest, ice, and bracing) and supervised physical therapy. Exclusion criteria included: inability to comply with follow-up, hematologic conditions, knee pain from other etiologies (e.g., degenerative joint disease, trauma), full or partial patellar tendon tear, use of anticoagulation or immunosuppression, prior surgery, pregnancy, worker’s compensation injury, daily opioid use, MRI contraindication, systemic diseases such as diabetes or connective tissue diseases and prior PRP or NT procedure.

Clinical outcome, US, and UTE MRI measures

VAS during activity, PT-specific Victorian Institute of Sport Assessment Patella (VISA-P) score and Tegner Activity Scale were collected at baseline, 16 weeks, and 52 weeks.^22,23^

Both conventional (B-mode and Power-doppler) and quantitative (SWE) US images were obtained by a single operator (KL) at all timepoints using a linear high frequency (6-15 MHz) transducer (Supersonic Imagine, Aix-en-Provence, France) with a gel stand-off pad in long axis to the patella tendon with the knee in 30° flexion as measured by a goniometer.

A sagittal three-dimensional multi-echo UTE-T2* sequence (3D-Cones, GE Healthcare, Waukesha, WI) of the knee was performed at all time points using a 3.0T scanner (Discovery MR750, GE Healthcare, Waukesha, WI) and 8-channel phased-array extremity coil (InVivo, Orlando, FL). The sequence utilized a 3D cone k-space sampling scheme with 16 echo times between 0.003ms and 35ms. Additional parameters included a 40ms repetition time, 20° flip angle, 16cm field-of-view, ±150KHz bandwidth, 256×256 matrix, 3mm slice thickness, one excitation, 10 slices through the patellar tendon, and 19-minute scan time.

Image post-processing and analysis

A musculoskeletal radiologist with 5 years of experience (ZS) retrospectively reviewed all conventional and SWE US images. The thickest portion of the tendon (mm), and hypo-echogenicity and hyperemia (0=normal to 3=severe) were reported. The SWS maps had three 10 mm diameter regions of interest (ROIs) corresponding to diseased areas on the baseline B-mode images (Figure 1). SWS was averaged across the ROIs. Care was taken to measure SWS in the exact same location on follow-up.

For MRI, a musculoskeletal radiologist with 6 years of experience (RvdH) delineated ROIs of the proximal 1 cm of the patellar tendon on TE 4.3ms UTE image using 3D Slicer (Figure 2). All echoes were registered using rigid registration.^24^ Single-component T2*_single_ and bicomponent fast-relaxing macromolecular-bound water component (T2*_fast_), slow relaxing bulk water component (T2*_slow_) and fraction of fast-relaxing macromolecular-bound water component (F_F_) parameter maps were calculated by voxel-by-voxel curve fitting using MATLAB (MATLAB 2010b, MathWorks Inc, Natick, MA).^21^ After superimposing the ROI over the UTE-T2* parameter maps, mean and SD values were obtained. Excellent repeatability has been demonstrated in a prior study.^13^

Statistics

The sample size was calculated for comparing VISA-P score change from baseline to 52-weeks between groups. With an expected Cohen’s D effect size of 0.52, 20 subjects per group was estimated to result in a 95% power. With 29 total subjects, prior to analysis, there would be 65% power to detect an effect size of 0.52 and 80% power to detect a slight larger, but still attainable, effect size of 0.61. All analyses followed intention-to-treat. Missing data were treated as missing at random.

Longitudinal data analysis (LDA) with subject as a random effect were used to compare between group and within group differences in mean outcome measures over time. Group allocation, a 3-level factor for time, and their interaction term were fixed predictors in the LDA models. Pearson’s correlation was used to assess correlations of change from baseline to 52-weeks between outcome measures. To account for multiple comparisons, a Tukey’s family-wise adjustment was done in the case of a post-hoc examination of the 2-way comparison. P-values <0.05 were considered significant. All statistical analyses were performed in R v4.0.

**Results**

Study population

897 patients were screened, and ultimately 29 athletes were enrolled between April 2017 and July 2022 and randomized into the following groups: PRP=9; NT=11; and SH=9 (Figure 3, Table 1). One patient in the NT group did not receive the intervention because the patient did not qualify per imaging criteria for PT. One patient from the NT group was lost to follow-up. For the MRI analyses, one patient in the SH group was excluded due to mid-tendon involvement rather than proximal tendon. Three patients (1x PRP, 2x NT) were excluded from the MRI analysis for varying technical reasons such as failed registration and artifacts. MRI data was available for 19 patients at baseline (6 SH, 5 NT, 8 PRP), 20 patients at 16-week (7 SH, 7 NT, 6 PRP), and for 16 patients at 52-week (6 SH, 6 NT, 4 PRP). No adverse events were reported.

The patients in the three treatment arms were not statistically different with respect to age, BMI, sex, and race. Most participants were Caucasian.

Outcome

The PRP and SH groups demonstrated significant improvements in VAS pain during activity at 16-week (Table 2). At 52-week all groups demonstrated significant improvements in VAS pain during activity with this effect appearing most pronounced in the PRP group (Table 2). At 16-week, the VISA-P score improved significantly for the PRP group (20.3, 95%CI [5.0, 35.7], p=0.006). At 52-week, the VISA-P score improved statistically significantly for the PRP and SH groups (Table 2). On the Tegner Activity Scale, only the PRP group demonstrated a significant improvement at 52-week (Table 2).

Conventional US and SWE measurements

At baseline, the NT group had a significantly smaller tendon thickness (p = .027) and higher SWS (p = .025) than the SH group. The baseline conventional and qualitative US parameters were otherwise not significantly different amongst the treatment groups at baseline. At 16-week there was no significant change in the US parameters in any of the treatment groups. At 52-week, only the SH group experienced a significant change in the conventional US parameters with a decrease in both tendon thickness and hyperemia. There was no change in the hypo-echogenicity in any of the treatment groups at 52-week (Table 3). There was a significant increase in SWS in only the PRP group at 52-week (Table 4, Figure 4). The change in conventional US measures was not significantly correlated with the change in VAS (Table 5). The change in SWS at 52-week was moderately correlated with the change in VAS (-0.52 [-0.76, -0.15] p= .009) (Figure 5).

UTE MRI measurements

The NT group had a significantly lower T2*_fast_ (p = .027) compared to the SH group at baseline (Table 3). The baseline quantitative MRI parameters were otherwise not significantly different amongst the treatment groups. F_F_ significantly increased from baseline to 52-week in all groups (Figure 6). Only the PRP group demonstrated a significant decrease in T2*_Single_ (Figure 6). There was no significant change in T2*_fast_ or T2*_slow_ in any of the groups at 52-week. The changes in T2*_single_ and F_F_ at 52-week were moderately correlated with the change in VAS, respectively, but the p-values did not show statistical significance for these correlations (Table 5, Figure 5).

No correlations were demonstrated between either UTE measures or SWS and VISA-P. Also, no significant correlation was demonstrated between quantitative MRI parameters and SWS. F_F_ was significantly correlated with a decrease in hyperemia (-0.62 [-0.88, -0.07] p=.030).

Subgroup analysis

A total of 5 patients were affected by temporary (~5 month) restriction of team sport activities relating to the COVID-19 pandemic.^25^ Of these 5 patients, 3 were in the SH cohort, and there was 1 patient from both the NT and PRP cohorts. Subgroup analysis demonstrated that those unaffected by a temporary activity restriction related to the pandemic experienced a less dramatic decrease in VAS pain at 52-week (Supplemental table 1). This effect was primarily seen in the SH and NT cohorts and was negligible for the PRP cohort. In this sub-analysis, the improvement in pain in the NT group was not statistically significant, the SH group maintained a statistically significant improvement with smaller effect size, and the effect in the PRP groups was essentially unchanged with a significant reduction in VAS pain at 52-week.

**Discussion**

The presented sham-controlled RCT has compared PRP to NT using multimodal imaging to assess tendon healing over 52-weeks. Improvement in VAS was demonstrated across all treatment groups at 52-weeks; however, only the PRP group demonstrated significant improvement in all PROs. There was a moderate correlation between the changes in SWS and VAS at 52-weeks across treatment groups. Only the PRP group showed a significant decrease in T2*_single_ and increase in SWS at 52-weeks, while all groups demonstrated an increase in F_F_ at 52-weeks.

A key finding was the improvement in VAS across all treatment groups, including SH, which suggests improvement without any minimally invasive treatment.^27^ Activity restrictions imposed by the COVID-19 pandemic may have contributed to improvement in pain especially in the sham group, because the lockdown affected a larger proportion of subjects in that group. The placebo effect could also have contributed to these results.^28^

Among all treatment groups, the PRP cohort reported the greatest improvement in VAS and only the PRP cohort reported significant clinical improvement in all PROs at 52-weeks. The role of PRP in PT and its relative effectiveness to other interventions are still unclear. A 2022 meta-analysis found that PRP injections did not provide clinical benefit in terms of pain relief compared to other non-PRP injection alternatives.^29^ Additionally, a 2021 systematic review and meta-analysis concluded that there was no “high-quality evidence to convincingly demonstrate superior outcomes with specific treatment modalities (for patella tendinopathy)”.^30^

Current conventional qualitative imaging does not always correlate with clinical outcomes. Prior studies have suggested that ultrasound SWE may serve as a quantitative imaging metric to assess tendon healing.^17,18,31^ In this study, increase in SWS was correlated with improvement in PT-related pain, adding further credence to this notion. Interestingly, no significant correlation was found between the changes in SWS and the other PROs.

With regards to UTE T2* parameters, F_F,_ increased significantly across all groups at 52-weeks, while T2*_single_ only decreased significantly in the PRP group and no significance was demonstrated for T2*_fast_, T2*_slow_ . The significant changes in F_F_ were driven by both a decrease in bulk water and an increase in macromolecular bound water due to macromolecular matrix repair during tendon healing.^32^ The difference between single- and bi-component UTE is probably because T2*_single_ , a composite measure of bicomponent parameters T2*_fast_, T2*_slow_ and the weighting factor fraction of bound water F_F_ , is less specific to changes in the individual water compartments . These results were in concordance with prior literature showing that the fraction of free and bound water was more sensitive than T2*_single_, T2*_fast_, and T2*_slow_ for detecting compositional and structural changes in articular cartilage degeneration^33^ and tendinopathy*.*^13^

There were moderate correlations between VAS at 52-weeks and T2*_single_ and F_F_, respectively, but p-values were above the threshold for statistical significance. Other studies have reported correlations between PROs and UTE-MRI.^12^ It is possible that the results in this study were limited by the small sample size.

There was no correlation between SWS and F_F,_ or T2*_single_. This may be due to a lack of power or due to a true difference between imaging parameters. SWS reflects tendon elastic properties which is primarily dependent upon collagen fiber organization and macrostructure, while UTE-MRI may be more reflective of tendon biochemical composition and microstructure.^12,34^ F_f_ may be a more sensitive measure of clinical improvement—since an increased F_F_ was seen across all groups, just as improvement in VAS was. SWS could be a direct reflection of more robust or organized healing—since significant changes in SWS were only seen in the PRP group at 52-weeks. Quantitative US and MRI may serve as complementary, rather than interchangeable, modalities.

This study has several limitations. The COVID-19 pandemic limited recruitment and may have affected the participants’ activity level, indirectly affecting their clinical outcomes, especially in the sham group. Subgroup analysis of participants not being affected by temporary restriction of team sport activities, which may not be adequately powered, has been performed. The study was also limited by the small sample size and inability to acquire a substantial number of UTE-MRI follow-up data points. This precluded statistical adjustments for confounders and the powering necessary to find statistical significance for the correlations between the UTE-MRI parameters and PROs. The missing UTE-MRI data was equally distributed across groups, and the missing data points were utilized rather than imputing a large percentage of the data. A single operator performed all SWS US measurements. This was by design, as previous studies have shown good intra-rater reliability while inter-rater reliability is less reliable.^35,36^ This design, however, did limit the ability to perform any inter-rater reliability measures for SWS. Finally, the study was limited by demographic homogeneity.

In summary, this study suggests that improvement in pain may be the natural course of PT with rest and physical therapy. However, the greatest degree of clinical improvement was with PRP treatment when compared to SH and NT. Additionally, this study demonstrated that quantitative imaging may have a role as an adjunct measure of tendon healing. Most notably was the correlation between improvement in pain and an increase in SWS. Potential has been shown for UTE imaging, but studies with a larger sample size are needed to further validate its clinical applicability. The promising results of this study may help form the basis for future larger and more diverse clinical trials necessary to elucidate the clinical role of emerging minimally invasive treatments and quantitative imaging tools.

**References**

1. Ferretti A, Ippolito E, Mariani P, Puddu G. Jumper’s knee. *Am J Sports Med*. 1983;11(2):58-62. doi:10.1177/036354658301100202

2. Zwerver J, Mc Auliffe S, Rio EK, Scott A, Vicenzino BT, Weir A. ICON 2019-international scientific tendinopathy symposium: building an ICONic tendon tower-launching a new era in clinical tendinopathy research. *Br J Sports Med*. 2020;54(8):442-443. doi:10.1136/bjsports-2019-101214

3. Fredberg U, Stengaard-Pedersen K. Chronic tendinopathy tissue pathology, pain mechanisms, and etiology with a special focus on inflammation. *Scand J Med Sci Sports*. 2008;18(1):3-15. doi:10.1111/j.1600-0838.2007.00746.x

4. De Mos M, Van El B, DeGroot J, et al. Achilles Tendinosis: Changes in Biochemical Composition and Collagen Turnover Rate. *Am J Sports Med*. 2007;35(9):1549-1556. doi:10.1177/0363546507301885

5. Xu Y, Murrell GAC. The basic science of tendinopathy. *Clin Orthop*. 2008;466(7):1528-1538. doi:10.1007/s11999-008-0286-4

6. Riley G. The pathogenesis of tendinopathy. A molecular perspective. *Rheumatol Oxf Engl*. 2004;43(2):131-142. doi:10.1093/rheumatology/keg448

7. Stewart ZE, Lee K. Lower extremity ultrasound-guided interventions: tendon, ligament, and plantar fascia. *Skeletal Radiol*. 2023;52(5):991-1003. doi:10.1007/s00256-022-04212-4

8. Docking SI, Ooi CC, Connell D. Tendinopathy: Is Imaging Telling Us the Entire Story? *J Orthop Sports Phys Ther*. 2015;45(11):842-852. doi:10.2519/jospt.2015.5880

9. Crawford SK, Thelen D, Yakey JM, Heiderscheit BC, Wilson JJ, Lee KS. Regional shear wave elastography of Achilles tendinopathy in symptomatic versus contralateral Achilles tendons. *Eur Radiol*. 2023;33(1):720-729. doi:10.1007/s00330-022-08957-3

10. Dirrichs T, Quack V, Gatz M, et al. Shear Wave Elastography (SWE) for Monitoring of Treatment of Tendinopathies: A Double-blinded, Longitudinal Clinical Study. *Acad Radiol*. 2018;25(3):265-272. doi:10.1016/j.acra.2017.09.011

11. Liu J, Nazaran A, Ma Y, et al. Single- and Bicomponent Analyses of T2 ⁎ Relaxation in Knee Tendon and Ligament by Using 3D Ultrashort Echo Time Cones (UTE Cones) Magnetic Resonance Imaging. *BioMed Res Int*. 2019;2019:1-9. doi:10.1155/2019/8597423

12. Loegering IF, Denning SC, Johnson KM, Liu F, Lee KS, Thelen DG. Ultrashort echo time (UTE) imaging reveals a shift in bound water that is sensitive to sub-clinical tendinopathy in older adults. *Skeletal Radiol*. 2021;50(1):107-113. doi:10.1007/s00256-020-03538-1

13. Kijowski R, Wilson JJ, Liu F. Bicomponent ultrashort echo time T2* analysis for assessment of patients with patellar tendinopathy. *J Magn Reson Imaging JMRI*. 2017;46(5):1441-1447. doi:10.1002/jmri.25689

14. Juras V, Apprich S, Szomolanyi P, Bieri O, Deligianni X, Trattnig S. Bi-exponential T2* analysis of healthy and diseased Achilles tendons: an in vivo preliminary magnetic resonance study and correlation with clinical score. *Eur Radiol*. 2013;23(10):2814-2822. doi:10.1007/s00330-013-2897-8

15. Breda SJ, de Vos RJ, Poot DHJ, Krestin GP, Hernandez-Tamames JA, Oei EHG. Association Between T2 * Relaxation Times Derived From Ultrashort Echo Time MRI and Symptoms During Exercise Therapy for Patellar Tendinopathy: A Large Prospective Study. *J Magn Reson Imaging JMRI*. 2021;54(5):1596-1605. doi:10.1002/jmri.27751

16. Pauli C, Bae WC, Lee M, et al. Ultrashort-echo time MR imaging of the patella with bicomponent analysis: correlation with histopathologic and polarized light microscopic findings. *Radiology*. 2012;264(2):484-493. doi:10.1148/radiol.12111883

17. Quilling GM, Lee KS, Ebben B. Shear wave elastography imaging in a porcine tendinopathy model. *Skeletal Radiol*. 2022;51(11):2167-2173. doi:10.1007/s00256-022-04073-x

18. Aubry S, Risson JR, Kastler A, et al. Biomechanical properties of the calcaneal tendon in vivo assessed by transient shear wave elastography. *Skeletal Radiol*. 2013;42(8):1143-1150. doi:10.1007/s00256-013-1649-9

19. Lee KS, Martin J, Thelen D. Science to Practice: Quantitative US Elastography Can Be Used to Quantify Mechanical and Histologic Tendon Healing in a Rabbit Model of Achilles Tendon Transection. *Radiology*. 2017;283(2):311-313. doi:10.1148/radiol.2017170126

20. Chang EY, Du J, Chung CB. UTE imaging in the musculoskeletal system. *J Magn Reson Imaging JMRI*. 2015;41(4):870-883. doi:10.1002/jmri.24713

21. Liu F, Kijowski R. Assessment of different fitting methods for in-vivo bi-component T2* analysis of human patellar tendon in magnetic resonance imaging. *Muscles Ligaments Tendons J*. 2017;7(1):163-172. doi:10.11138/mltj/2017.7.1.163

22. Visentini PJ, Khan KM, Cook JL, Kiss ZS, Harcourt PR, Wark JD. The VISA score: an index of severity of symptoms in patients with jumper’s knee (patellar tendinosis). Victorian Institute of Sport Tendon Study Group. *J Sci Med Sport*. 1998;1(1):22-28. doi:10.1016/s1440-2440(98)80005-4

23. Tegner Y, Lysholm J. Rating systems in the evaluation of knee ligament injuries. *Clin Orthop*. 1985;(198):43-49.

24. Klein S, Staring M, Murphy K, Viergever MA, Pluim JPW. elastix: a toolbox for intensity-based medical image registration. *IEEE Trans Med Imaging*. 2010;29(1):196-205. doi:10.1109/TMI.2009.2035616

25. Sabbagh RS, Shah NS, Kanhere AP, Hoge CG, Thomson CG, Grawe BM. Effect of the COVID-19 Pandemic on Sports-Related Injuries Evaluated in US Emergency Departments. *Orthop J Sports Med*. 2022;10(2):232596712210753. doi:10.1177/23259671221075373

26. Dragoo JL, Wasterlain AS, Braun HJ, Nead KT. Platelet-rich plasma as a treatment for patellar tendinopathy: a double-blind, randomized controlled trial. *Am J Sports Med*. 2014;42(3):610-618. doi:10.1177/0363546513518416

27. Rosen AB, Wellsandt E, Nicola M, Tao MA. Clinical Management of Patellar Tendinopathy. *J Athl Train*. 2022;57(7):621-631. doi:10.4085/1062-6050-0049.21

28. Miller FG, Colloca L, Kaptchuk TJ. The placebo effect: illness and interpersonal healing. *Perspect Biol Med*. 2009;52(4):518-539. doi:10.1353/pbm.0.0115

29. Barman A, Sinha MK, Sahoo J, et al. Platelet-rich plasma injection in the treatment of patellar tendinopathy: a systematic review and meta-analysis. *Knee Surg Relat Res*. 2022;34(1):22. doi:10.1186/s43019-022-00151-5

30. Challoumas D, Pedret C, Biddle M, et al. Management of patellar tendinopathy: a systematic review and network meta-analysis of randomised studies. *BMJ Open Sport Exerc Med*. 2021;7(4):e001110. doi:10.1136/bmjsem-2021-001110

31. DeWall RJ, Slane LC, Lee KS, Thelen DG. Spatial variations in Achilles tendon shear wave speed. *J Biomech*. 2014;47(11):2685-2692. doi:10.1016/j.jbiomech.2014.05.008

32. Thomopoulos S, Parks WC, Rifkin DB, Derwin KA. Mechanisms of tendon injury and repair. *J Orthop Res Off Publ Orthop Res Soc*. 2015;33(6):832-839. doi:10.1002/jor.22806

33. Liu F, Choi KW, Samsonov A, et al. Articular Cartilage of the Human Knee Joint: In Vivo Multicomponent T2 Analysis at 3.0 T. *Radiology*. 2015;277(2):477-488. doi:10.1148/radiol.2015142201

34. Kubo K, Kawakami Y, Fukunaga T. Influence of elastic properties of tendon structures on jump performance in humans. *J Appl Physiol Bethesda Md 1985*. 1999;87(6):2090-2096. doi:10.1152/jappl.1999.87.6.2090

35. Ito N, Sigurðsson HB, Pohlig RT, Cortes DH, Grävare Silbernagel K, Sprague AL. Reliability of Continuous Shear Wave Elastography in the Pathological Patellar Tendon. *J Ultrasound Med*. 2023;42(5):1047-1055. doi:10.1002/jum.16115

36. Taş S, Onur MR, Yılmaz S, Soylu AR, Korkusuz F. Shear Wave Elastography Is a Reliable and Repeatable Method for Measuring the Elastic Modulus of the Rectus Femoris Muscle and Patellar Tendon. *J Ultrasound Med*. 2017;36(3):565-570. doi:10.7863/ultra.16.03032

**Tables**

**Table 1 Patient characteristics**

|  | **Total** | **PRP** | **Needle tenotomy** | **Sham** | **p-value** |
| --- | --- | --- | --- | --- | --- |
| **N** | 29 | 9 | 11 | 9 |  |
| **Age** *mean (sd)* | 26.1 (5.3) | 25.7 (4.9) | 26.6 (5.6) | 25.8 (5.9) | 0.908 |
| **BMI** *mean (sd)* | 26.4 (5.0) | 26.9 (5.8) | 25.2 (2.9) | 27.4 (6.3) | 0.604 |
| **Sex (M: F)** | 24 (83%) : 5 (17%) | 8 (89%) :1 (11%) | 9 (82%): 2 (18%) | 7 (78%): 2 (22%) | 1 |
| **Race (self-reported)** |  |  |  |  |  |
| African-American (black) | 1 | 1 (11.1%) | 0 (0.0%) | 0 (0.0%) |  |
| American Indian or Alaska  Native | 1 | 0 (0.0%) | 0 (0.0%) | 1 (11.1%) |  |
| Caucasian | 27 | 8 (88.9%) | 11 (100.0%) | 8 (88.9%) |  |

PRP=platelet rich plasma

**Table 2 Clinical outcome at baseline, week 16 and week 52 across the three treatment groups.**

|  | **Week 0** | **Week 16** | **Week 52** | **Week 16-0** | **P-value** | **Week 52-0** | **P-value** |
| --- | --- | --- | --- | --- | --- | --- | --- |
| **VAS** (0 - 10) | | | | | | | |
| PRP | 7.4 (5.9, 9.0) | 4.1 (2.5, 5.6) | 1.6 (0.0, 3.2) | -3.39 (-5.29, -1.49) | **< 0.001** | -5.87 (-7.84, -3.89) | **<.001** |
| NT | 6.9 (5.5, 8.3) | 5.7 (4.2, 7.2) | 4.5 (2.8, 6.1) | -1.24 (-3.09, 0.62) | 0.26 | -2.45 (-4.47, -0.43) | **.01** |
| SH | 7.7 (6.1, 9.2) | 4.4 (2.9, 6.0) | 3.3 (1.8, 4.9) | -3.22 (-5.13, -1.32) | **< 0.001** | -4.33 (-6.24, -2.43) | **<.001** |
| *group-level* | p = .39 |  |  |  |  |  | **.04** |
| **VISA-P** (0 - 100) | | | | | | | |
| PRP | 45.0 (32.2, 57.8) | 65.3 (52.5, 78.1) | 76.8 (64.0, 89.6) | 20.3 (5.0, 35.7) | **0.006** | 31.8 (16.4, 47.1) | **<.001** |
| NT | 57.9 (46.3, 69.5) | 69.4 (56.9, 82.0) | 69.3 (55.6, 83.0) | 11.5 (-3.46, 26.5) | 0.17 | 11.4 (-4.9, 27.7) | .23 |
| SH | 56.0 (43.2, 68.8) | 66.6 (53.7, 79.4) | 73.7 (60.3, 87.0) | 10.6 (-4.78, 25.9) | 0.24 | 17.7 (1.7, 33.6) | **.03** |
| *group-level* | p = .23 |  |  |  |  |  | .15 |
| **Tegner Activity Scale (1**-10) | | | | | | | |
| PRP | 4.0 (2.8, 5.2) | 4.7 (3.5, 6.0) | 6.1 (4.8, 7.3) | 0.72 (-0.71, 2.15) | 0.478 | 2.06 (0.63, 3.48) | **.003** |
| NT | 6.1 (5.0, 7.2) | 5.7 (4.5, 6.9) | 6.6 (5.2, 7.9) | -0.38 (-1.77, 1.02) | 0.82 | 0.47 (-1.06, 1.99) | .78 |
| SH | 6.6 (5.3, 7.8) | 5.9 (4.6, 7.1) | 6.4 (5.2, 7.7) | -0.67 (-2.10, 0.76) | 0.54 | -0.11 (-1.54, 1.32) | .99 |
| *group-level* | PRP vs NT **p = .04**; PRP vs SH **p = .02** |  |  |  |  |  | .32 |

Reported as estimated mean (95% CI) from longitudinal data analysis with subject as a random effect. PRP=platelet rich plasma, NT=needle tenotomy, SH=sham, VISA-P = PT-specific Victorian Institute of Sport Assessment Patella, VAS=Visual analogue scale.

**Table 3 Conventional ultrasound measurements for baseline, week 16, and week 52 across the three treatment groups.**

|  | **Week 0** | **Week 16** | **Week 52** | **Week 16-0** | **P-value** | **Δ Week 52-0** | **P-value** |
| --- | --- | --- | --- | --- | --- | --- | --- |
| **Thickness** | | | | | | | |
| PRP | 7.6 (6.6, 8.6) | 7.5 (6.5, 8.5) | 7.0 (6.0, 8.0) | -0.16 (-0.96, 0.65) | 0.86 | -0.64 (-1.45, 0.16) | .14 |
| NT | 6.2 (5.2, 7.1) | 6.4 (5.4, 7.3) | 6.4 (5.4, 7.4) | 0.20 (-0.60, 1.00) | 0.78 | 0.21 (-0.67, 1.09) | .80 |
| SH | 8.0 (7.0, 9.0) | 8.1 (7.0, 9.1) | 6.7 (5.7, 7.7) | 0.08 (-0.73, 0.89) | 0.95 | -1.30 (-2.11, -0.49) | **.001** |
| *group-level* | NT vs SH **p = .03** |  |  |  |  | PRP vs SH **p = .02** | .**04** |
| **Echotexture** | | | | | | | |
| PRP | 1.9 (1.3, 2.5) | 1.9 (1.3, 2.5) | 1.9 (1.3, 2.5) | 0.0 (-0.64, 0.64) | 1 | -8.1 (-14.6, -1.6) | 1 |
| NT | 2.0 (1.5, 2.5) | 1.8 (1.3, 2.4) | 2.0 (1.4, 2.6) | -0.17 (-0.80, 0.46) | 0.76 | -0.01 (-0.70, 0.68) | .99 |
| SH | 2.0 (1.4, 2.6) | 2.4 (1.9, 3.0) | 1.7 (1.1, 2.3) | 0.44 (-0.20, 1.09) | 0.21 | -0.33 (-0.98, 0.31) | .40 |
| *group-level* | p = .95 |  |  |  |  |  | .193 |
| **Hyperemia** | | | | | | | |
| PRP | 1.8 (1.1, 2.5) | 1.3 (0.7, 2.0) | 1.1 (0.4, 1.8) | -0.44 (-1.12, 0.23) | 0.24 | -0.67 (-1.34, 0.01) | .053 |
| NT | 1.2 (0.6, 1.8) | 1.1 (0.4, 1.7) | 0.5 (-0.2, 1.2) | -0.10 (-0.76, 0.57) | 0.91 | -0.67 (-1.40, 0.06) | .07 |
| SH | 2.1 (1.4, 2.8) | 2.0 (1.3, 2.7) | 1.1 (0.4, 1.8) | -0.11 (-0.79, 0.56) | 0.89 | -1.00 (-1.67, -0.33) | **.003** |
| *group-level* | P = .13 |  |  |  |  |  | .583 |

Reported as estimated mean (95% CI) from longitudinal data analysis with subject as a random effect. PRP=platelet rich plasma, NT=needle tenotomy, SH=sham.

**Table 4 Quantitative imaging values on** ultrashort TE MRI and ultrasound elastography **for baseline, week 16, and week 52 across the three treatment groups.**

|  | **Week 0** | **Week 16** | **Week 52** | **Week 16-0** | **P-value** | **Δ Week 52-0** | **P-value** |
| --- | --- | --- | --- | --- | --- | --- | --- |
| **T2*_single_ (ms)** | | | | | | | |
| PRP | 15.2 (11.8, 18.6) | 11.7 (7.7, 15.7) | 7.1 (2.3, 12.0) | -3.5 (-9.2, 2.2) | .28 | -8.1 (-14.6, -1.6) | **.010** |
| NT | 10.6 (6.3, 15.0) | 8.7 (5.0, 12.4) | 6.9 (2.9, 10.9) | -1.9 (-8.2, 4.3) | .68 | -3.7 (-10.2, 2.8) | .32 |
| SH | 14.9 (11.0, 18.9) | 13.4 (9.7, 17.1) | 11.7 (7.7, 15.7) | -1.6 (-7.5, 4.3) | .75 | -3.2 (-9.3, 2.9) | .37 |
| *group-level* | p = .47 |  |  |  |  |  | .75 |
| **F_F_ (%)** | | | | | | | |
| PRP | 0.5 (0.5, 0.6) | 0.6 (0.5, 0.6) | 0.6 (0.5, 0.7) | 0.02 (-0.06, 0.11) | .74 | 0.10 (0.00, 0.20) | **.046** |
| NT | 0.6 (0.5, 0.6) | 0.6 (0.5, 0.7) | 0.7 (0.6, 0.7) | 0.03 (-0.07, 0.12) | .75 | 0.11 (0.01, 0.21) | **.030** |
| SH | 0.4 (0.4, 0.5) | 0.5 (0.4, 0.6) | 0.5 (0.5, 0.6) | 0.07 (-0.02, 0.16) | .17 | 0.10 (0.01, 0.20) | **.020** |
| *group-level* | p = .13 |  |  |  |  |  | .89 |
| **T2*_fast_ (ms)** | | | | | | | |
| PRP | 2.2 (1.7, 2.7) | 1.9 (1.3, 2.5) | 2.1 (1.4, 2.8) | -0.26 (-1.02, 0.50) | .64 | -0.06 (-0.95, 0.82) | .97 |
| NT | 1.8 (1.1, 2.4) | 2.1 (1.5, 2.7) | 2.6 (2.0, 3.2) | 0.33 (-0.52, 1.18) | .57 | 0.82 (-0.07, 1.71) | .07 |
| SH | 2.9 (2.3, 3.5) | 2.6 (2.0, 3.2) | 2.5 (2.0, 3.1) | -0.27 (-1.06, 0.53) | .65 | -0.32 (-1.14, 0.49) | .55 |
| *group-level* | NT vs SH **p=.03** |  |  |  |  |  | .27 |
| **T2*_slow_ (ms)** | | | | | | | |
| PRP | 30.0 (24.6, 35.3) | 27.6 (21.4, 33.7) | 28.6 (21.2, 36.1) | -2.4 (-10.6, 5.8) | .71 | -1.3 (-10.8, 8.2) | .91 |
| NT | 27.9 (21.2, 34.6) | 28.8 (23.0, 34.5) | 33.2 (27.1, 39.4) | 0.8 (-8.3, 9.9) | .96 | 5.3 (-4.2, 14.9) | .34 |
| SH | 28.4 (22.2, 34.6) | 29.2 (23.5, 35.0) | 28.6 (22.5, 34.8) | 0.8 (-7.7, 9.4) | .95 | 0.2 (-8.6, 9.0) | .96 |
| *group-level* | p = .92 |  |  |  |  |  | .73 |
| **SWS (m/s)** | | | | | | | |
| PRP | 6.3 (5.1, 7.4) | 7.3 (6.1, 8.4) | 8.6 (7.4, 9.7) | 1.0 (-0.56, 2.56) | .26 | 2.31 (0.75, 3.88) | **.003** |
| NT | 7.6 (6.5, 8.6) | 7.5 (6.3, 8.6) | 7.0 (5.7, 8.3) | -0.09 (-1.60, 1.42) | .98 | -0.60 (-2.24, 1.05) | .62 |
| SH | 5.7 (4.6, 6.9) | 5.9 (4.7, 7.0) | 6.4 (5.2, 7.6) | 0.12 (-1.44, 1.69) | .97 | 0.67 (-0.90, 2.23) | .52 |
| *group-level* | NT vs SH **p = .03** |  |  |  |  | PRP vs NT **p = .01** | .08 |

Reported as estimated mean (95% CI) from longitudinal data analysis with subject as a random effect. PRP=platelet rich plasma, NT=needle tenotomy, SH=sham. T2*single =single-component UTE-T2* relaxation time, F_F_ =fraction of the fast-relaxing macromolecular bound water component, T2*fast =fast-relaxing macromolecular-bound water component, T2*slow= slow relaxing bulk water component, SWS=shear wave speed on ultrasound

| **Table 5 Linear Correlation of change from baseline to week 52 for quantitative MRI and ultrasound measures, conventional ultrasound measures and patient reported outcomes, .**   \|  \| **Variable** \| **Correlation (95% CI)** \| **P value** \| \| --- \| --- \| --- \| --- \| \| **T2* single** \| \| \| \| \|  \| Thickness \| 0.08 (-0.52, 0.62) \| .81 \| \|  \| Echotexture \| -0.17 (-0.68, 0.45) \| .61 \| \|  \| Hyperemia \| 0.06 (-0.53, 0.62) \| .84 \| \|  \| SWS \| -0.39 (-0.79, 0.24) \| .22 \| \|  \| VAS Pain \| 0.53 (-0.11, 0.86) \| .10 \| \|  \| VISA-P \| 0.07 (-0.55, 0.64) \| .83 \| \| **Ff** \| \| \| \| \|  \| Thickness \| -0.19 (-0.69, 0.43) \| .56 \| \|  \| Echotexture \| -0.30 (-0.75, 0.33) \| .34 \| \|  \| Hyperemia \| **-0.62 (-0.88, -0.07)** \| **.030** \| \|  \| SWS \| 0.03 (-0.55, 0.60) \| .92 \| \|  \| VAS Pain \| -0.51 (-0.85, 0.13) \| .11 \| \|  \| VISA-P \| 0.04 (-0.58, 0.62) \| .91 \| \| **T2* fast** \| \| \| \| \|  \| Thickness \| 0.19 (-0.43, 0.69) \| .56 \| \|  \| Echotexture \| -0.44 (-0.81, 0.18) \| .15 \| \|  \| Hyperemia \| -0.18 (-0.68, 0.44) \| .58 \| \|  \| SWS \| -0.54 (-0.85, 0.04) \| .07 \| \|  \| VAS Pain \| 0.44 (-0.21, 0.82) \| .17 \| \|  \| VISA-P \| 0.18 (-0.47, 0.70) \| .60 \| \| **T2* slow** \| \| \| \| \|  \| Thickness \| -0.03 (-0.59, 0.55) \| .93 \| \|  \| Echotexture \| -0.46 (-0.82, 0.16) \| .13 \| \|  \| Hyperemia \| -0.46 (-0.82, 0.15) \| .13 \| \|  \| SWS \| -0.36 (-0.77, 0.27) \| .26 \| \|  \| VAS Pain \| 0.29 (-0.38, 0.76) \| .40 \| \|  \| VISA-P \| 0.20 (-0.46, 0.71) \| .56 \| \| **SWS** \| \| \| \| \|  \| Thickness \| -0.39 (-0.68, 0.00) \| .053 \| \|  \| Echotexture \| -0.13 (-0.50, 0.28) \| .52 \| \|  \| Hyperemia \| -0.03 (-0.42, 0.37) \| .88 \| \|  \| VAS Pain \| **-0.52 (-0.76, -0.15)** \| **.009** \| \|  \| VISA-P \| 0.09 (-0.32, 0.48) \| .67 \| |  |
| --- | --- | --- | --- | --- | --- | --- | --- | --- | --- | --- | --- | --- | --- | --- | --- | --- | --- | --- | --- | --- | --- | --- | --- | --- | --- | --- | --- | --- | --- | --- | --- | --- | --- | --- | --- | --- | --- | --- | --- | --- | --- | --- | --- | --- | --- | --- | --- | --- | --- | --- | --- | --- | --- | --- | --- | --- | --- | --- | --- | --- | --- | --- | --- | --- | --- | --- | --- | --- | --- | --- | --- | --- | --- | --- | --- | --- | --- | --- | --- | --- | --- | --- | --- | --- | --- | --- | --- | --- | --- | --- | --- | --- | --- | --- | --- | --- | --- | --- | --- | --- | --- | --- | --- | --- | --- | --- | --- | --- | --- | --- | --- | --- | --- | --- | --- | --- | --- | --- | --- | --- | --- | --- | --- | --- | --- | --- | --- | --- | --- | --- | --- | --- | --- | --- | --- | --- | --- | --- | --- | --- | --- |
| Reported as Pearson correlation coefficient and 95% CI for change from baseline at Week 52. T2*single =single-component UTE-T2* relaxation time, F_F_ =fraction of the fast-relaxing macromolecular bound water component, T2*fast =fast-relaxing macromolecular-bound water component, T2*slow= slow relaxing bulk water component, SWS = Shear wave speed on ultrasound, VISA-P = PT-specific Victorian Institute of Sport Assessment Patella, VAS=Visual analogue scale. |  |

**Figures and figure legends**

Figure 1. Example of the delineated region of interest (ROI) of the proximal patellar tendon on ultrasound grey scale and corresponding shear wave speed (SWS) color-map images.


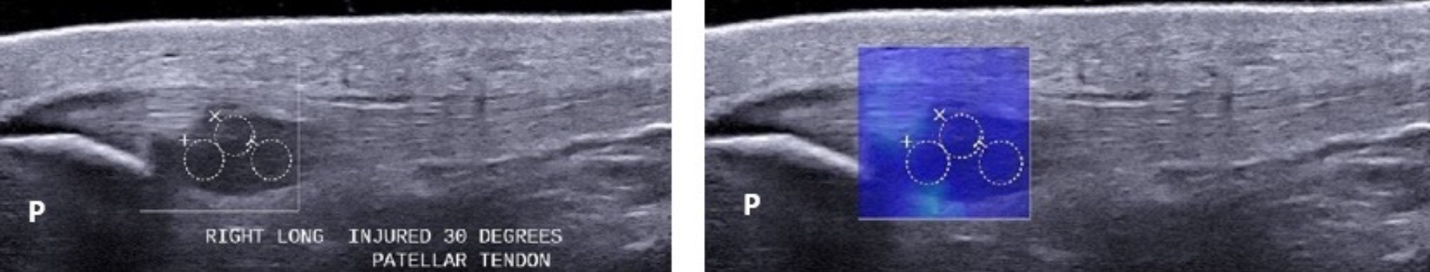


Figure 2. Example of the delineated region of interest (ROI) of the proximal patellar tendon on a ultrashort TE-T2* MR image.


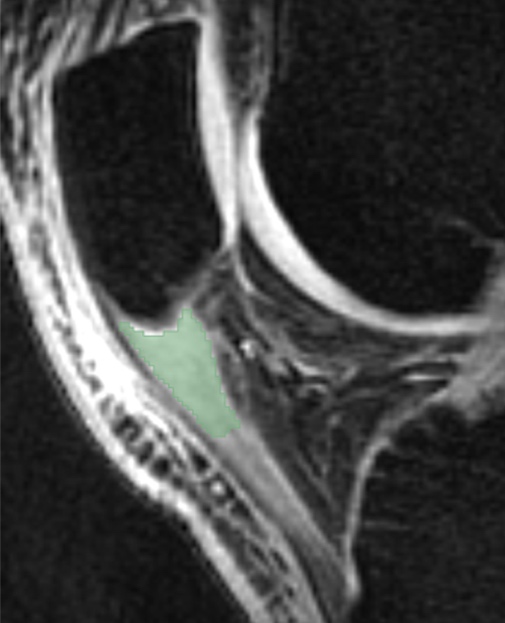


Figure 3. Flowchart of patient inclusion.

PRP=platelet rich plasma, NT=needle tenotomy, SH=sham

Figure 4. Sagittal ultrasound shear wave speed (SWS) maps at baseline (left) and 52-week follow-up (right) in three patients with patellar tendinopathy (26 year-old male, 25 year-old male, 20 year-old female) who underwent platelet rich plasma (PRP), needle tenotomy and sham treatment respectively. After PRP treatment statistically significant improvement in SWS from 6.3 to 10.8m/s was demonstrated, while the needle tenotomy and sham treatment showed no improvement over time.


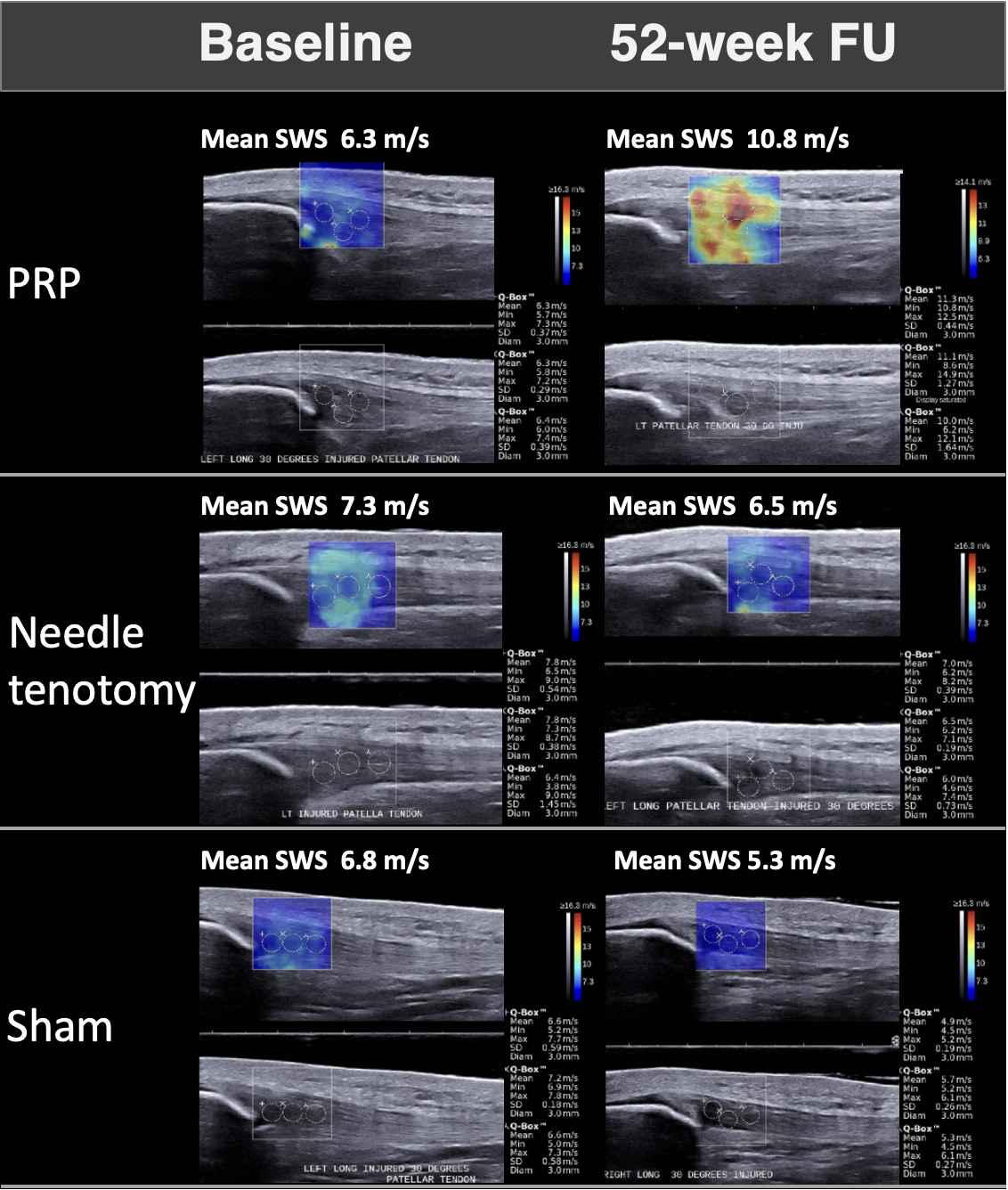


Figure 5. Plots showing correlations between change in visual analogue scale (VAS) pain score and changes in imaging values on ultrashort TE MRI and ultrasound elastography at week 52.


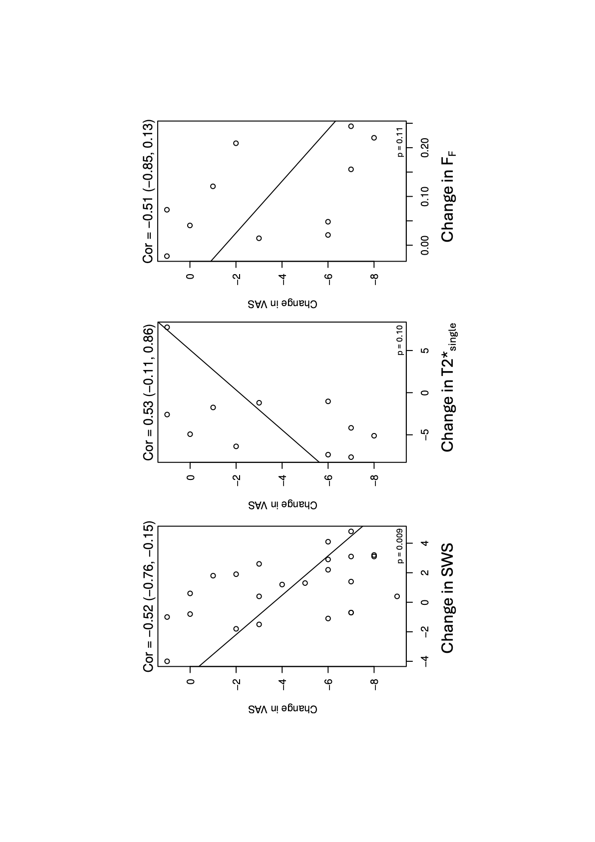


T2*single =single-component UTE-T2* relaxation time, F_F_ =fraction of the fast-relaxing macromolecular bound water component, SWS=shear wave speed on ultrasound

Figure 6. MRI T2*_single_ (ms) and F_F_ (%) parameter maps at baseline and 52-week follow-up in three patients with patellar tendinopathy (30 year-old male, 34 year-old male, 20 year-old male) following platelet rich plasma (PRP), needle tenotomy, and sham treatment respectively. Improvements in T2*_single_ and F_F_ were seen in all groups, but the greatest improvement was demonstrated in the PRP group. T2*single =single-component UTE-T2* relaxation time, F_F_ =fraction of the fast-relaxing macromolecular bound water component


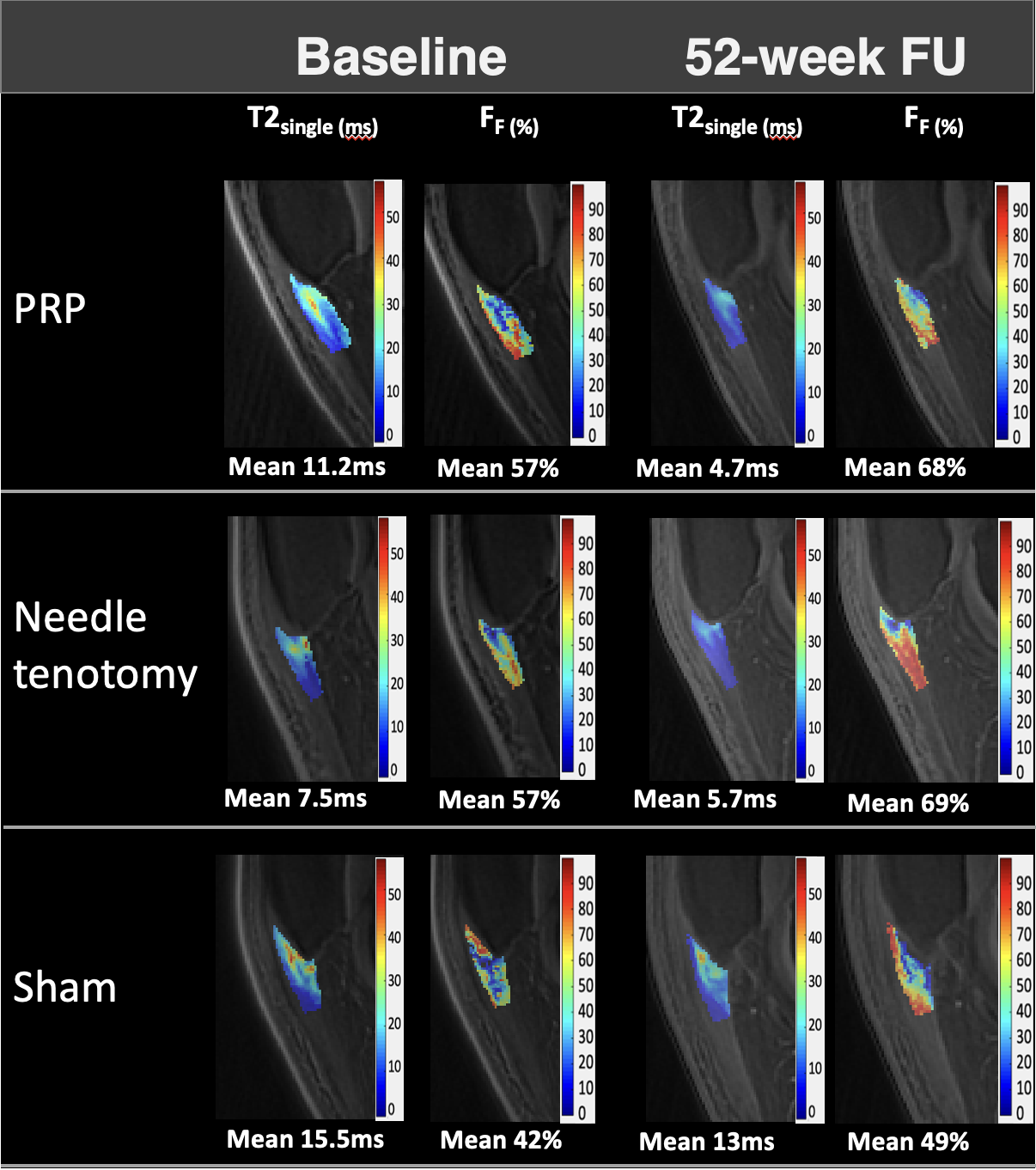


**Supplements**

**Supplemental table 1 – Clinical outcome at baseline, week 16, and week 52 across the three treatment groups for only those patients that were unaffected by temporary restriction of team sport activities.**

Reported as estimated mean (95% CI) from longitudinal data analysis with subject as a random effect. PRP=platelet rich plasma, NT=needle tenotomy, SH=sham, VISA-P = PT-specific Victorian Institute of Sport Assessment Patella, VAS=Visual analogue scale.

|  | **Week 0** | **Week 16** | **Week 52** | **Week 16-0** | **P-value** | **Week 52-0** | **P-value** |
| --- | --- | --- | --- | --- | --- | --- | --- |
| **VAS** (0 - 10) | | | | | | | |
| PRP | 7.3 (5.7, 8.8) | 4.2 (2.6, 5.8) | 1.5 (-0.1, 3.2) | -3.06 (-4.86, -1.26) | < 0.001 | -5.70 (-7.58, -3.82) | < 0.001 |
| NT | 6.8 (5.4, 8.2) | 5.4 (3.9, 7.0) | 4.9 (3.2, 6.6) | -1.39 (-3.15, 0.36) | 0.15 | -1.86 (-3.80, 0.08) | 0.06 |
| SH | 7.7 (5.8, 9.5) | 4.7 (2.8, 6.5) | 4.8 (3.0, 6.7) | -3.00 (-5.08, -0.92) | 0.003 | -2.83 (-4.91, -0.76) | 0.005 |
| *group-level* | p = .36 |  |  |  |  |  | 0.007 |
| **VISA-P** (0 - 100) | | | | | | | |
| PRP | 46.6 (32.7, 60.5) | 67.4 (53.5, 81.3) | 76.7 (62.9, 90.6) | 20.75 (3.89, 37.61) | 0.012 | 30.12 (13.26, 46.99) | < 0.001 |
| NT | 58.9 (46.4, 71.4) | 69.5 (55.8, 83.1) | 67.9 (52.8, 83.1) | 10.60 (-5.81, 27.00) | 0.29 | 9.03 (-9.08, 27.15) | 0.48 |
| SH | 55.5 (39.4, 71.6) | 65.2 (49.0, 81.3) | 72.6 (55.4, 89.8) | 9.67 (-9.81, 29.14) | 0.49 | 17.08 (-3.63, 37.80) | 0.13 |
| *group-level* | p = .36 |  |  |  |  |  | 0.25 |
| **Tegner Activity Scale** (1-10) | | | | | | | |
| PRP | 3.9 (2.6, 5.1) | 5.1 (3.8, 6.3) | 5.7 (4.4, 6.9) | 1.19 (-0.18, 2.55) | 0.1 | 1.81 (0.45, 3.18) | 0.006 |
| NT | 5.7 (4.6, 6.8) | 5.4 (4.2, 6.6) | 6.3 (5.0, 7.6) | -0.34 (-1.67, 1.00) | 0.84 | 0.63 (-0.85, 2.10) | 0.6 |
| SH | 6.0 (4.6, 7.4) | 5.7 (4.2, 7.1) | 6.3 (4.9, 7.8) | -0.33 (-1.91, 1.24) | 0.89 | 0.33 (-1.24, 1.91) | 0.89 |
| *group-level* | p = .04; no sig. 2-way comparison after adjustment |  |  |  |  |  | 0.25 |
